# Antihypertensive therapy for pregnancy hypertension and implications for fetal and neonatal heart rate monitoring: A systematic review of randomized trials and observational studies

**DOI:** 10.1111/aogs.70019

**Published:** 2025-08-20

**Authors:** Akalya Ganeshamoorthy, Omiete Fubara Duke, Hiten D. Mistry, Jeffrey N. Bone, Marianne Vidler, Edgardo Abalos, Katie Badawy, Asma Khalil, Peter von Dadelszen, Laura A. Magee

**Affiliations:** ^1^ Department of Women and Children's Health King's College London London UK; ^2^ Clinical Research Support Unit British Columbia Children's Hospital Research Institute Vancouver British Columbia Canada; ^3^ Department of Obstetrics and Gynaecology University of British Columbia Vancouver British Columbia Canada; ^4^ Department of Obstetrics Maternity Martín Hospital Rosario Santa Fe Argentina; ^5^ Faculty of Life Sciences and Medicine King's College London London UK; ^6^ Molecular & Clinical Sciences Research Institute St. George's, University of London London UK

**Keywords:** antihypertensive, fetal heart rate, neonatal heart rate, observational study, pregnancy, randomized trial

## Abstract

**Introduction:**

Our objective was to evaluate whether antihypertensives affect fetal (FHR) or neonatal (neoHR) heart rate.

**Material and methods:**

Electronic databases and clinical trial registers were searched to August 31, 2024. Eligibility included randomized (RCTs) or observational studies evaluating antihypertensives for pregnancy hypertension. Two reviewers independently assessed studies for inclusion and extracted data. Random effects meta‐analysis was used to determine risk ratios (RRs) and 95% confidence intervals (CIs). Network meta‐analysis was undertaken in a sensitivity analysis.

**Results:**

Fifty‐four RCTs (*n* = 5736 pregnancies) and 28 observational studies (*n* = 2 283 855) reported FHR (usually visually‐interpreted) or neoHR (usually clinically‐assessed).

**FHR: Non‐Severe Hypertension:**

Antihypertensives did not increase adverse FHR effects in RCTs of antihypertensives versus placebo/no therapy (RR = 1.08, 95% CI [0.62–1.89]; *I* ^2^ = 43%; *N* = 10, *n* = 1567 pregnancies), antihypertensives versus methyldopa (RR = 1.40 [0.97–2.04]; *I* ^2^ = 0%; *N* = 6, *n* = 515), or labetalol or pure beta‐blockers versus other antihypertensives (RR = 1.70 [0.96–2.99]; *I* ^2^ = 30%; *N* = 5, *n* = 501). In observational studies, adverse FHR effects were more common with: labetalol versus methyldopa, nifedipine or Chinese herbal medication (RR = 2.17 [1.15–4.08]; *I* ^2^ = 47%; *N* = 4, *n* = 664), and bendroflumethiazide versus metoprolol (but not hydralazine), but 95% CIs were wide.

**FHR: Severe Hypertension:**

Antihypertensives had no FHR effects in RCTs of antihypertensives versus either: placebo/no therapy (RR = 0.43 [0.16–1.20]; *I* ^2^ = 0%; *N* = 3, *n* = 242), hydralazine (RR = 0.71 [0.29–1.72]; *I* ^2^ = 13%; *N* = 11, *n* = 727), or CCBs (RR = 0.52 [0.12–2.16]; *I* ^2^ = 0%, *N* = 9, *n* = 1675). In observational studies, there was no difference for labetalol versus other antihypertensives (RR = 0.34 [0.10–1.14], *I* ^2^ = 87%; *N* = 4, *n* = 590), with heterogeneity due to a lower‐quality labetalol versus hydralazine study. There were fewer adverse FHR effects for nifedipine versus hydralazine study (RR = 0.09 [0.01–0.68]; *n* = 49).

**NeoHR: Severe Hypertension:**

RCTs of antihypertensives versus placebo/no therapy were not associated with adverse neoHR effects (RR = 1.26 [0.31–5.19]; *I* ^2^ = 66%; *N* = 4, *n* = 406), with heterogeneity attributed to more neoHR effects with continuously monitored neoHR. Observational studies revealed no effect on neoHR of antihypertensives versus no therapy (RR = 1.06 [0.67–1.67]; *I* ^2^ = 54%; *N* = 4, *n* = 37 359), but labetalol was associated with more adverse effects and metoprolol with fewer. In RCTs of antihypertensives versus other antihypertensives, there was no difference in adverse neoHR (RR = 3.0 [0.13–71.74]; *N* = 3, *n* = 162). Observational studies showed adverse neoHR effects in labetalol versus pure beta‐blockers (RR = 1.99 [1.36–2.91]; *I* ^2^ = 0%; *N* = 3, *n* = 16 204). No severe hypertension RCTs reported neoHR. Observational studies were limited. Network meta‐analysis showed no significant relationships between antihypertensives and FHR or neoHR; 95% CIs were very wide.

**Conclusions:**

Evidence is inadequate to draw reliable conclusions about the impact of antihypertensives on FHR or neoHR. At present, adverse FHR or neoHR effects should be attributed to evolving placental dysfunction.

## Graphical abstract

Trials are mostly reassuring regarding antihypertensive effects on fetal (FHR) or neonatal heart rate (neoHR), but observational studies raise concerns about beta‐blockers, particularly the alpha/beta‐blocker labetalol. Clinicians should ascribe adverse fetal or neonatal heart rate effects to evolving underlying placental dysfunction or newborn illness, respectively, and not to antihypertensive therapy.

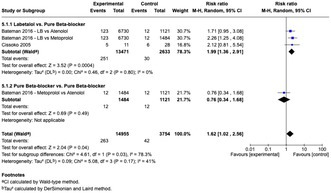

**Key message**

Evidence is inadequate to draw reliable conclusions about the impact of antihypertensives on fetal or neonatal heart rate. Adverse effects should be attributed to evolving placental dysfunction.

## 1 INTRODUCTION

Collectively, the hypertensive disorders of pregnancy (HDPs) complicate up to 10% of pregnancies, globally,^1^ and are a major cause of maternal and fetal/newborn complications at preterm and term gestational ages.^2^ Hypertension in pregnancy is defined as a consistent systolic blood pressure (BP) ≥ 140 mmHg or diastolic BP ≥ 90 mmHg.^3^

The care of women with HDPs is multifaceted. Monitoring of maternal well‐being involves clinical assessment and laboratory testing for end‐organ dysfunction. Fetal well‐being is evaluated by fetal heart rate (FHR) and pattern, and ultrasonographic assessment of fetal growth and resistance to flow through major vessels (i.e., fetal Doppler). Timed birth is recommended at term gestational age for women with pre‐eclampsia and is considered at any gestational age when assessment of maternal or fetal well‐being is not reassuring. Antihypertensive therapy is recommended at various BP thresholds, usually 140/90 mmHg and at minimum for chronic hypertension, to reduce the incidence of severe hypertension and the severe features (maternal end‐organ complications) of pre‐eclampsia, without increasing the risk of fetal growth restriction.^4^, ^5^

A number of antihypertensive medications are commonly used in pregnancy: oral labetalol, calcium channel blockers (CCBs, particularly nifedipine), and methyldopa for non‐severe hypertension, and for severe hypertension, most guidelines advise use of parenteral labetalol, oral nifedipine, or parenteral hydralazine.^6^ All of these medications are known to cross the human placenta, but without evidence of fetal accumulation.^7^, ^8^

FHR assessment is a mainstay of fetal assessment, as HR is an indicator of fetal oxygenation status and well‐being. Although it has been shown in some animal models that antihypertensives may affect fetal physiology, including FHR,^9^, ^10^, ^11^ our 2004 systematic review by Waterman et al found that available data were insufficient to support concern,^12^ although they were of limited quality and most studies used methods of FHR assessment that are subject to bias (i.e., visual interpretation of cardiotocography [CTG]). Nevertheless, there are courses on intrapartum CTG interpretation that teach that antihypertensive therapy can alter FHR and pattern.^13^ Also, antihypertensive medication may have neonatal health effects, through drug in the fetal circulation at birth (that must be cleared by the newborn) and/or drug contained in breastmilk.

We aimed to update the previous systematic review of the impact on FHR or neonatal HR (neoHR) of antihypertensive therapy in hypertensive pregnancy.

## 2 MATERIAL AND METHODS

This systematic review was prospectively registered, and amendments documented (CRD42023459425). Ethical approval was not required, as the review involved secondary analysis of published research findings. The authors declare that all supporting data are available within the article and in online supplementary files.

### 2.1 Search strategy, information sources, and eligibility criteria

The review methods were based on guidelines developed by the Cochrane Collaboration,^14^ and designed to update our 2004 systematic review covering literature from 1975 to March 2001^12^—literature that was also included in this review.

A comprehensive search, developed in consultation with an information specialist, was undertaken of electronic databases, from January 1, 2001 to August 31, 2024 for randomized controlled trials (RCTs) or observational studies. The databases searched were Medline, PubMed, Embase, Cumulative Index to Nursing and Allied Health Literature (CINAHL), Cochrane Central Register of Controlled Trials (CENTRAL), and Web of Science. Additional searches were undertaken for randomized trials, in Clinical Trials.gov, the World Health Organization (WHO) International Clinical Trials Registry Platform (ICTRP), Latin American and Caribbean Health Sciences Literature (LILACS), and the Cochrane Pregnancy and Childbirth Trial register. The basic search terms were: “labetalol OR nifedipine OR methyldopa OR hydralazine” AND “pregnancy OR hypertension induced pregnancy OR preeclampsia” AND “neonatal OR fetal heart rate (FHR) OR non‐stress test OR cardiotocogram”; however, exact search terms were adapted to adhere to each database, as detailed in Table S1. Additional relevant trials were identified from a 2021 network meta‐analysis for treatment of non‐severe hypertension,^15^ and a 2018 systematic review of trials for treatment of severe hypertension.^16^ Language restrictions were not applied.

### 2.2 Study selection

Included were publications that: (i) were RCTs, quasi‐RCTs (analyzed as observational studies), controlled observational studies (if ≥50% of participants had an HDP, and our main outcome of interest was presented), or case series of at least six women; (ii) enrolled women with any form or severity of pregnancy hypertension; and (iii) examined the effects of antihypertensives administered on fetal or neonatal HR or pattern.

Hypertension was defined as: systolic BP ≥ 140 mmHg or diastolic BP ≥ 90 mmHg (including severe hypertension, defined as systolic BP ≥ 160 mmHg or diastolic BP ≥ 110 mmHg), or when authors described participants as having hypertension without specifying their BP values.

For single‐dose antihypertensive therapy, studies were included that examined outcomes within 24 h following medication administered orally, and within 6 h following parenteral medication.

Antihypertensives were any medications administered to lower BP; those of interest were labetalol, nifedipine, methyldopa, and hydralazine, regardless of the route of administration. Comparators were no medication, placebo, or another antihypertensive medication. For chronically‐administered antihypertensives, there was no specification for the timing of HR assessment. Studies were included regardless of prior antihypertensive treatment or multiple gestation. Studies reporting FHR or neoHR effects only as continuous variables were included for descriptive purposes.

Excluded were animal studies, those of interventions designed to prevent progression to pre‐eclampsia, and studies examining the effects of antihypertensives postpartum (unless the outcomes were reported separately for women treated antepartum). Trials that were retracted were not eligible.

The main outcomes were FHR and neoHR patterns, determined from any type of HR monitoring, including fetal visually‐interpreted and computerized CTG, and defined by the authors.

The secondary outcomes included other fetal monitoring ultrasonographic parameters (i.e., Doppler ultrasound of umbilical and middle cerebral arteries [MCA] or the ductus venosus; amniotic fluid index; deepest vertical pocket of amniotic fluid; and fetal biophysical profile), and the core outcomes in pregnancy hypertension^17^ for the mother (i.e., maternal mortality, eclampsia, stroke, cortical blindness, retinal detachment, pulmonary edema, acute kidney injury, liver capsule hematoma or rupture, placental abruption, postpartum hemorrhage, raised liver enzymes, low platelets, intubation and mechanical ventilation) and baby (i.e., stillbirth, neonatal mortality, gestational age at birth, birthweight [BW], small‐for‐gestational‐age [SGA] infants, newborn convulsions, neonatal respiratory support, and admission to a special care baby unit or neonatal intensive care unit).

### 2.3 Data extraction

Two reviewers (AG, OFD) independently assessed all abstracts, using Rayyan software.^18^ Full texts of relevant reports were retrieved and reviewed. Data abstraction, including assessment of Cochrane trustworthiness criteria,^19^ was undertaken by two reviewers independently (OFD, AG, or KB). Authors were contacted if there were uncertainties about published data or trustworthiness criteria; trials were classified as “awaiting classification” if no response was received from study authors, or reassurance was not provided regarding trustworthiness criteria. Disagreement was resolved by consulting a third reviewer (LAM, PvD, or HDM).

### 2.4 Assessment of risk of bias

Risk of bias was assessed by three standard quality assessment tools, as relevant.^20^, ^21^, ^22^ For RCTs, quality was designated as low, high, or unclear based on: random sequence generation, allocation concealment, blinding of participants and personnel, blinding of outcome assessment, incomplete outcome data, and selective reporting^22^; a trial was at high risk of bias overall if it were at high risk of bias in either random sequence generation or allocation concealment. For observational studies, the Newcastle‐Ottawa Scale (NOS) was used to grade quality based on eight criteria for study selection, comparability, and exposure.^20^ For case series, the Joanna Briggs Institute Checklist was used to evaluate the risk of bias, based on 10 criteria; scores of 8–10 reflected high quality, 5–7 moderate quality, and <5 low quality.^21^

### 2.5 Data synthesis

Descriptive analysis was undertaken of the characteristics of the studies, participants, intervention, and outcomes of FHR or neoHR. For each outcome, the impact of antihypertensive medication was compared with placebo/no therapy and with other antihypertensives. Results were summarized with risk ratios (RRs) and 95% confidence intervals (CIs), using a random effects model. Heterogeneity was quantified using the I^2^ statistic and interpreted as: <40% “might not be important,” 30–60% “may represent moderate heterogeneity,” 50–90% “may represent substantial heterogeneity,” and ≥75% representing “considerable heterogeneity”.^14^

Publication bias for main outcomes was assessed when there were ≥10 informative trials. Funnel plots were used to assess asymmetry visually and if asymmetry was suggested, an exploratory analysis was planned to investigate it via the Egger test or the trim‐and‐fill method.

A similar approach was planned for other fetal monitoring ultrasonographic parameters. For other pregnancy (secondary) outcomes, median event rates were calculated for context.

Two sensitivity analyses were undertaken for FHR and neoHR effects: (i) excluding RCTs and quasi‐randomized trials if authors had citations on Retraction Watch; and (ii) performing a network meta‐analysis (NMA) to consider, for each of non‐severe and severe hypertension treatment and the impact on adverse FHR and neoHR effects, the impact of all drugs versus placebo/no therapy or drug versus drug combinations. In the NMA, any two interventions are compared by combining direct estimates, obtained by pooling data from head‐to‐head studies that compared those interventions, while indirect estimates were obtained by pooling data from studies through all common comparators.^23^ Where possible, we decomposed effects into the direct and indirect components. In cases where the number of studies/events were too small for splitting, we only provided the overall combined estimates. We used a Bayesian random effects model with a *logit* link function, using the *getmc* package in R statistical software version 4.4.2.^24^

Meta‐analyses were conducted using the Cochrane Review Manager software (version 5.4). A 95% CI that excluded 1.0 was considered statistically significant.

## 3 RESULTS

### 3.1 Study selection

**FIGURE 1:**
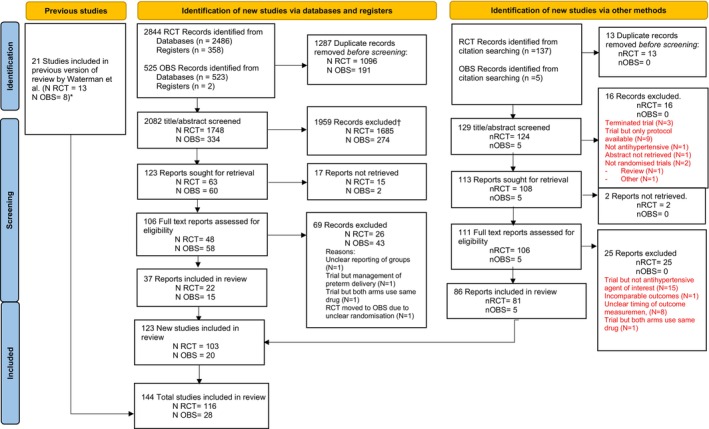
Preferred Reporting Items for Systematic Reviews and Meta‐Analyses 2020 flow diagram for updated systematic reviews which included searches of databases, registers, and other sources. *N*, number; OBS, observational; RCT, randomized controlled trial. *From studies in the Waterman et al. review, one RCT was excluded due to overlap, an two OBS studies were excluded due to wrong outcome and study design. †For a list of trials excluded, please see Table S2.

Figure 1 shows that in addition to 21 studies from the 2004 review on this topic,^12^ 3369 new studies (including 2844 trials and 525 observational studies) were identified through database and register searches, and 142 new studies (137 trials and 5 observational studies) were found from other sources. Following the removal of duplicate records, title and abstract screening, and full‐text review, 144 studies (116 trials and 28 observational studies) were included. Of the 116 trials, 62 did not report outcomes of interest and were not considered further (Table S2). As such, there were 82 studies (54 trials [5736 women] and 28 observational studies [2 283 855 women]) included in our main analysis.

Table 1 presents the study and participant characteristics of the 82 included studies (2 289 591 participants), with full details of each study presented in Tables S3 and S4.

**TABLE 1:** Study characteristics (*N* (%) or median [IQR] unless otherwise specified).

	RCTs		Observational studies	
	Non‐severe hypertension (*N* = 30)	Severe hypertension (*N* = 24)	Non‐severe hypertension (*N* = 19)	Severe hypertension (*N* = 9)
Study characteristics				
Study type				
Retrospective	NA	NA	13 (68.4%)	3 (33.3%)
Prospective	30 (100%)	24 (100%)	3 (15.7%)	2 (22.2%)
Quasi‐randomized	NA	NA	3 (15.7%)	4 (44.4%)
Centre status				
Single center	25 (83.3%)	19 (79.2%)	17 (89.4%)	9 (100%)
Multiple centers <5	2 (6.7%)	5 (20.8%)	0	0
Multiple centers ≥5	3 (10%)	0	2 (10.5%)	0
LMIC	7 (23.3%)	20 (83.3%)	5 (26.3%)	1 (11.1%)
Funding reported	11 (36.7%)	8 (33.3%)	2 (22.2%)	1 (11.1%)
Full publication	30 (100%)	24 (100%)	19 (100%)	9 (100%)
Includes unpublished data	2 (6.7%)	2 (8.3%)	0	0
Risk of bias assessment				
Cochrane RoB tool	*N* = 27	*N* = 24	*N* = 3, Quasi‐RCTs	*N* = 4, Quasi‐RCTs
Low risk of bias	3 (10%)	4 (16.7%)	0	0
High risk of bias	0	0	1 (33.3%)	2 (50%)
Unclear risk of bias	27 (90%)	20 (83.3%)	2 (66.7%)	2 (50%)
Newcastle Ottawa Scale			*N* = 12	*N* = 3
Good quality	NA	NA	5 (41.6%)	2 (66.7%)
Fair quality	NA	NA	7 (58.3%)	1 (33.3%)
Poor quality	NA	NA	0	0
Joanna Briggs checklist			*N* = 4	*N* = 2
High quality	NA	NA	4 (100%)	2 (100%)
Moderate quality	NA	NA	0	0
Low quality	NA	NA	0	0
Retraction Watch citation	2 (6.7%)	2 (8.3%)	0	0
Participants	*N* = 3393	*N* = 2343	*N* = 2 282 911^a^	*N* = 944
*N*/study	95 [35, 139.5]	55 [27.5, 110.8]	47 (20, 114)	100 (49, 109)
GA at enrolment (weeks)				
First trimester	1 (3.3%)	0	0	0
Second trimester	2 (6.7%)	0	1 (5.2%)	0
Third trimester	15 (50%)	23 (95.8%)	10 (52.6%)	6 (66.7%)
Unspecified	2 (6.7%)	0	0	0
Multiple gestation groups	10 (33.3%)	1 (4.2%)	8 (42.1%)	3 (33.3%)
Type of hypertension				
Chronic hypertension	3 (10%)	0	0	0
Gestational hypertension	0	1 (4.2%)	5 (26.3%)	2 (22.2%)
PIH	6 (20%)	0	0	0
Pre‐eclampsia	4 (13.3%)	3 (2.5%)	1 (5.2%)	3 (33.3%)
Mixed	10 (33.3%)	4 (16.7%)	10 (52.6%)	3 (33.3%)
Unspecified	7 (23.3%)	16 (66.7%)	4 (21%)	1 (11.1%)
Hypertension severity				
Non‐severe	30 (100%)	0	19 (100%)	0
Severe	0	24 (100%)	0	9 (100)
Both	0		0	
Demographics				
Maternal age reported	24/30 (80%)	22/24 (91.7%)	7 (36.8%)	6 (66.6%)
Age (year)	27.4 [24.0, 29.0]	26.8 [25.3, 29.6]	29.5 [27.2, 31.7]	28.6 [27.4, 30.65]
BMI (kg/m^2^) reported	1/30 (3.3%)	3/24 (12.5%)	2 (10.5%)	1 (11.1%)
BMI (kg/m^2^)	Not reported^b^	30.9 [27.4, 34.1]	29.88 [25.3, 35]	31.45 (1 study)
Smoking reported	10/30 (33.3%)	1/24 (4.2%)	2 (10.5%)	0
Smokers	15.0%	3.9%	10.5%	0
Antihypertensive used^c^				
Labetalol	12 (40%)	17 (70.8%)	11 (57.8%)	5 (55.5%)
Pure beta‐blocker	11 (36.7%)	0	7 (36.8%)	0
Methyldopa	15 (50%)	1 (4.2%)	5 (26.3%)	1 (11.1%)
Calcium channel blocker	5 (16.7%)	16 (66.7%)	3 (15.7%)	3 (33.3%)
Hydralazine	5 (16.7%)	13 (4.2%)	2 (10.5%)	4 (44.4%)
Others^d^	2 (6.7%)	0	0	0
Administration route				
Oral only	27 (90%)	3 (8.3%)	16 (84.2%)	3 (33.3%)
Parenteral only	2 (6.7%)	10 (41.7%)	3 (15%)	5 (55.5%)
Oral and parenteral	1 (3.3%)	11 (45.8%)	0	1 (11.1%)
Unspecified	0	0	0	0
Outcome (HR)				
FHR studies	21 (70%)^e^	24 (100%)	8 (42.1%)	8 (88.9%)
Neonatal HR studies	10 (30%)^e^	0	11 (57.8%)	1 (11.1%)
FHR monitoring method	(*N* = 21)	(*N* = 24)	(*N* = 8)	(*N* = 7)
Visual interpretation	12/21 (57.1%)	6/24 (25.0%)	6/8 (75.0%)	7/7 (100%)
Computerized CTG	1/21 (4.8%)	0	0	0
Fetal scalp electrode	1/21 (4.8%)	0	0	0
Not specified	7/21 (33.3%)	18/24 (75.0%)	2/8 (25.0%)	0
Neonatal HR monitoring method	*N* = 10	*N* = 0	*N* = 11	*N* = 1
Intermittent auscultation	2/10 (20.0%)	NA	3/11 (27.3%)	1 (100%)
Continuous	1/10 (10.0%)	NA	2/11 (18.2%)	0
Not specified	7/10 (70.0%)	NA	6/11 (54.5%)	0

*Note*: Some studies did not report adverse FHR or neonatal HR events as a binary event, but rather, as a continuous HR.

Abbreviations: CTG, cardiotocogram; FHR, fetal heart rate; GA, gestational age; HR, heart rate; IQR, interquartile range; LMIC, low‐ and middle‐income country; NA, not applicable; NICU, neonatal intensive care unit; PIH, pregnancy‐induced hypertension; RCTs, randomized controlled trials; RoB, risk of bias; SCBU, special care baby unit.

^a^ 1 study accounted for >95% of the total study population with 2 281 531 participants (41 018 informative participants) [Bateman et al.^66^].

^b^ This study reported that 24/63 (38%) of participants were obese, without reporting the BMI value [Lardoux et al.^42^].

^c^ The options are not mutually exclusive.

^d^ Other medicines given as antihypertensives were clonidine (given with hydralazine [Phippard et al.^36^]) and thiazide diuretics (given alongside either methyldopa or hydralazine [Arias and Zamora^50^]).

^e^ One trial examined both FHR and neonatal HR effects [Högstedt et al.^46^].

### 3.2 Study characteristics

Treatment of non‐severe hypertension was evaluated by 30 trials (3393 participants)^25^, ^26^, ^27^, ^28^, ^29^, ^30^, ^31^, ^32^, ^33^, ^34^, ^35^, ^36^, ^37^, ^38^, ^39^, ^40^, ^41^, ^42^, ^43^, ^44^, ^45^, ^46^, ^47^, ^48^, ^49^, ^50^, ^51^, ^52^, ^53^, ^54^ and 19 observational studies (2 282 911 participants).^55^, ^56^, ^57^, ^58^, ^59^, ^60^, ^61^, ^62^, ^63^, ^64^, ^65^, ^66^, ^67^, ^68^, ^69^, ^70^, ^71^, ^72^, ^73^ Treatment of severe hypertension was evaluated by 24 trials (2343 participants)^74^, ^75^, ^76^, ^77^, ^78^, ^79^, ^80^, ^81^, ^82^, ^83^, ^84^, ^85^, ^86^, ^87^, ^88^, ^89^, ^90^, ^91^, ^92^, ^93^, ^94^, ^95^, ^96^, ^97^ and 9 observational studies (944 participants)^98^, ^99^, ^100^, ^101^, ^102^, ^103^, ^104^, ^105^, ^106^ (Table 1). Almost all studies were from single centers. The minority were from low‐ and middle‐income countries other than for severe hypertension RCTs. A minority reported their funding source, and almost all were full publications. Observational studies were usually quasi‐randomized or retrospective.

### 3.3 Risk of bias of included studies

For RCTs, the risk of bias was usually unclear, most often because the method of randomization and nature of allocation concealment were not reported. For observational studies, study quality was generally good, other than for quasi‐randomized trials. Full details are presented in Table S5 (for RCTs and quasi‐randomized RCTs), Tables S6 (for controlled observational studies), and Table S7 (for case series). Four trials of treatment of non‐severe^44^, ^48^ or severe^75^, ^88^ hypertension had one or more authors with a citation (other than that included) on Retraction Watch.

In general, the number of participants was no greater than a median of 100, almost all of whom were enrolled later in pregnancy, particularly in their third trimester, meaning the duration of therapy was most often for weeks (Table 1). Most participants had a mixed or unspecified HDP type. Just over half of RCTs and most observational studies evaluated treatment of non‐severe hypertension. When maternal age was reported, most participants were in their late 1920s. Few studies of any type reported body mass index (BMI), the median of which was in the normal to overweight range. Many non‐severe hypertension trials reported smoking, practiced by a minority of women.

Most studies evaluated the impact of labetalol or a “pure” beta‐blocker on FHR or neoHR, although half of non‐severe hypertension RCTs and a number of observational studies evaluated methyldopa, and most severe hypertension RCTs and observational studies evaluated CCBs or hydralazine (Table 1). Oral administration of antihypertensives was undertaken in most non‐severe hypertension RCTs (except for one study that administered labetalol intravenously^41^) and observational studies. Parenteral administration of antihypertensives was undertaken in the majority of severe hypertension RCTs (except for oral nifedipine,^83^, ^91^ and one trial administering oral labetalol and oral methyldopa^88^).

Most studies evaluated the impact of antihypertensives on FHR, particularly in RCTs, whereas many observational studies reported the impact on neoHR. While many studies did not specify how FHR was assessed, when they did, visual interpretation of the CTG was the norm. One RCT (and no observational studies) used the computerized CTG. Most trials and observational studies did not report their method of neoHR monitoring. Details of other pregnancy outcomes reported are presented in Table S8.

### 3.4 Synthesis of results

#### 3.4.1 Adverse FHR effects

##### 3.4.1.1 Treatment of non‐severe hypertension

###### 3.4.1.1.1 Antihypertensive versus placebo/no therapy

**FIGURE 2:**
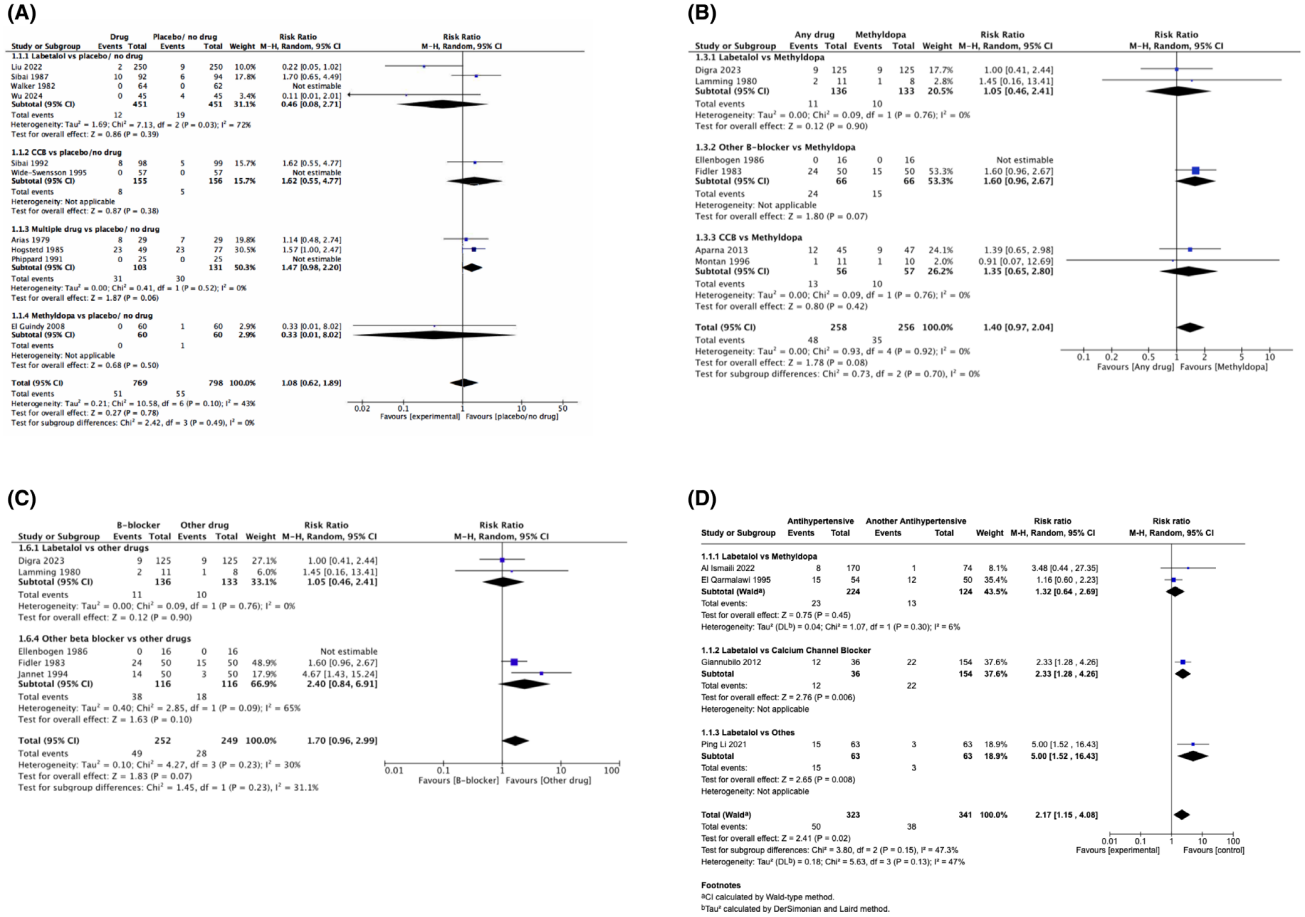
(A) Forest plots of RCTs assessing the impact of antihypertensives (vs. placebo/no therapy) on adverse FHR effects, among women treated for non‐severe hypertension. (B) Forest plots of RCTs assessing the impact of antihypertensives (vs. methyldopa) on adverse FHR effects among women treated for non‐severe hypertension. (C) Forest plots of RCTs assessing the impact of labetalol or other beta‐blockers (vs. other antihypertensives) on adverse FHR effects among women treated for non‐severe hypertension. (D) Forest plots of OBSERVATIONAL studies showing the effects of antihypertensives (vs. other antihypertensives) on adverse FHR effects among women with non‐severe hypertension. B‐blocker, beta‐blocker; CCB, calcium channel blocker; FHR, fetal heart rate; RCTs, randomized controlled trials.

In RCTs, antihypertensives (vs. placebo/no therapy) had no impact on adverse FHR effects (RR 1.08, 95% CI 0.62–1.89, *I* ^2^ = 43%; 6.6% versus 6.9%; 10 trials, 1567 pregnancies) (Figure 2A). The antihypertensives were labetalol,^27^, ^31^, ^41^, ^54^ CCBs (nifedipine^29^ or isradipine^25^), methyldopa,^48^ and multiple antihypertensives (either hydralazine and clonidine,^36^ hydralazine and metoprolol,^46^ or in another trial, either methyldopa and thiazide, hydralazine and thiazide, or methyldopa, hydralazine and thiazide^50^). The between‐trial heterogeneity was within the labetalol (vs. placebo/no therapy) trials; a potential explanation is that RCTs with RR <1.0 were conducted in China, while the RCT with RR >1.0 was from the United States. The results were unchanged in the first sensitivity analysis excluding papers with an author(s) cited on Retraction Watch, thereby excluding the trial by El Guindy et al. (RR 1.11, 95% CI 0.62–1.98, *I* ^2^ = 49%; Figure S1).^48^

In observational studies, no adverse FHR events were reported in antihypertensive versus no therapy groups (0/140 vs. 0/142; 4 studies with one reporting two comparison).^56^, ^57^, ^58^, ^73^ The antihypertensives were labetalol,^56^ methyldopa,^56^, ^73^ and nifedipine.^57^, ^58^

###### 3.4.1.1.2 Antihypertensive versus other agents

In RCTs, of antihypertensives (vs. other agents), there was no significant impact on adverse FHR effects, examined in two ways. First, antihypertensives were compared with methyldopa (RR 1.40, 95% CI 0.97–2.04, *I* ^2^ = 0%; 18.6% vs. 13.7%; 6 trials, 515 pregnancies) (Figure 2B); the antihypertensives were labetalol,^43^, ^49^ pure beta‐blockers (pindolol^47^ or oxprenolol^45^), and CCBs (nifedipine^51^ or isradipine^38^), and one trial contributed data to two subgroups.^90^ Second, labetalol or pure beta‐blockers were compared with other antihypertensives (RR 1.70, 95% CI 0.96–2.99, *I* ^2^ = 30%; 19.4% vs. 11.2%; 5 trials, 501 pregnancies) (Figure 2C); the other antihypertensives studied were methyldopa (as in Figure 2B, ^43^, ^45^, ^47^, ^49^) and nicardipine. Although the effect was not statistically significant, the data are consistent with a 4% reduction or 199% increase in adverse FHR effects, due possibly to “pure” beta‐blockers; Figure 2C results were unchanged in the Retraction Watch sensitivity analysis that excluded Jannet et al.^44^ (RR 1.42, 95% CI 0.92–2.20; Figure S2). In addition, three trials reported only the difference in mean FHR between antihypertensive groups^37^, ^39^, ^42^; a significant reduction in FHR was observed following atenolol (vs. pindolol) administration (−11.9 beats/min, *p* = 0.001),^39^ with “no difference” reported for metoprolol versus methyldopa,^37^ or labetalol versus acebutolol versus methyldopa.^42^

In observational studies of labetalol versus either methyldopa,^62^, ^67^ nifedipine,^63^ or a Chinese herbal remedy,^55^ labetalol appeared to increase adverse FHR effects (RR 2.17, 95% CI 1.15 to 4.08, I^2^ = 47%; 4 studies, 664 participants) (Figure 2D); potential heterogeneity appeared to be due to the labetalol (vs. methyldopa) comparison that found no effect, particularly the study that examined FHR in labor.^62^ In another study, bendroflumethiazide (with hydralazine administered to both groups) had more adverse FHR effects than metoprolol (16/97, 16.5% vs. 1/83, 1.2%; RR 13.69, 95% CI 1.85–101.05), but not hydralazine (with metoprolol administered to both groups; 7/101, 6.9% vs. 1/83, 1.2%; RR 5.75, 95% CI 0.72–45.82), but the 95% CI were very wide.^72^

###### 3.4.1.1.3 Network meta‐analysis

In the NMA sensitivity analysis, there were no significant overall differences in adverse FHR effects between antihypertensives or placebo/no therapy, but 95% credible intervals were very wide (Table S9).

##### 3.4.1.2 Treatment of severe hypertension

###### 3.4.1.2.1 Antihypertensive versus placebo/no therapy

**FIGURE 3:**
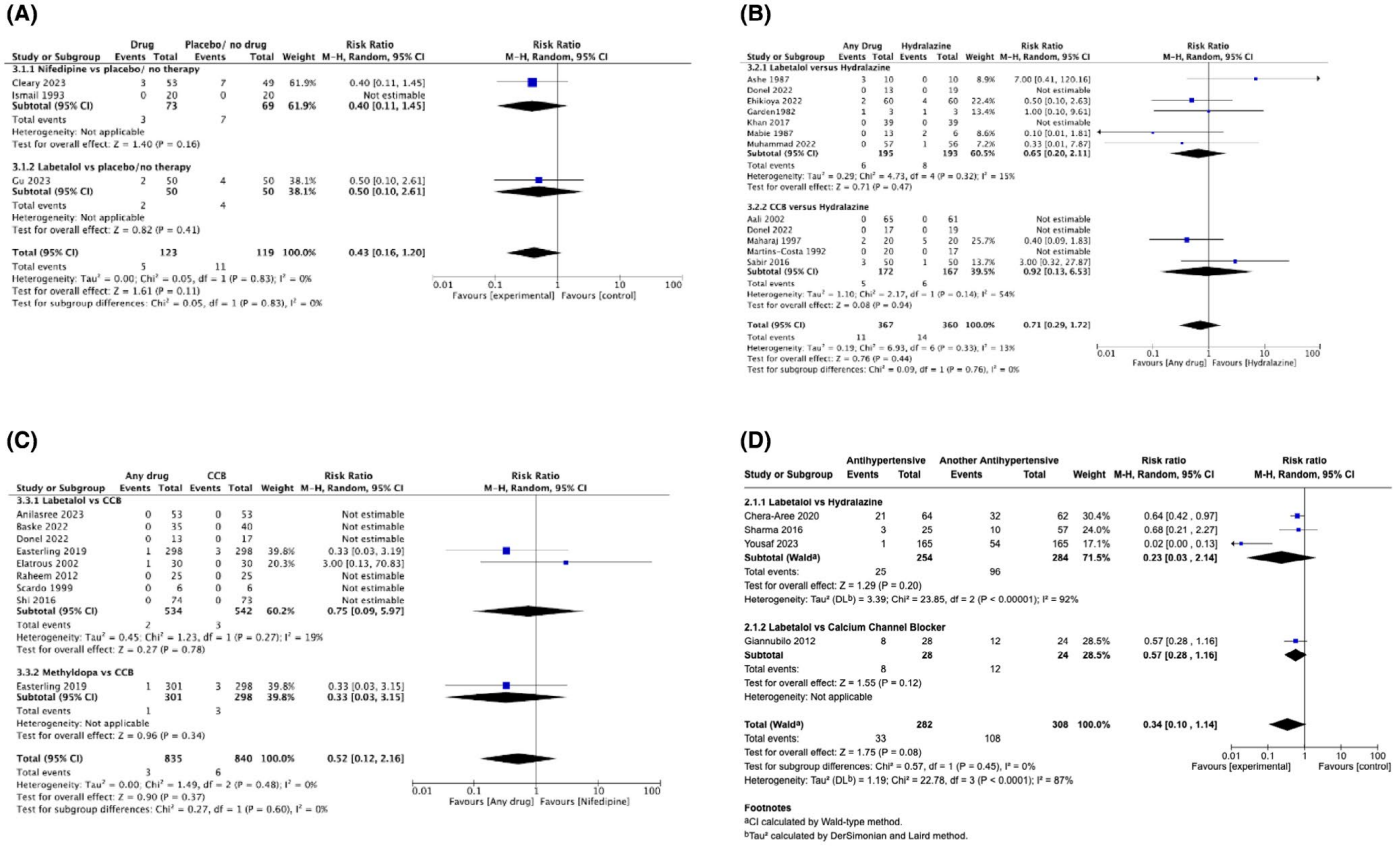
(A) Forest plots of RCTs evaluating the impact of antihypertensive therapy (vs. placebo/no treatment) on adverse FHR effects, among women with severe hypertension. (B) Forest plots of RCTs evaluating the impact of antihypertensive therapy (vs. hydralazine) on adverse FHR effects, among women with severe hypertension. (C) Forest plots of RCTs evaluating the impact of antihypertensive therapy (vs. CCBs) on adverse FHR effects, among women with severe hypertension. (D) Forest plots of OBSERVATIONAL studies showing the effects of antihypertensives on adverse FHR effects, among women with severe hypertension. CCB, calcium channel blocker; FHR, fetal heart rate; RCTs, randomized controlled trials.

In RCTs, antihypertensives (vs. placebo) had no impact on adverse FHR effects (RR 0.43, 95% CI 0.16 to 1.20, I^2^ = 0%; 4.1% vs. 9.2%; 3 trials; 242 participants) (Figure 3A). Antihypertensives included nifedipine (*N* = 2)^83^, ^91^ and labetalol (*N* = 1).^97^

In observational studies, there were no adverse FHR effects reported in antihypertensive versus no therapy groups (0/87 vs. 0/87; 3 studies).^100^, ^103^, ^104^ The antihypertensives evaluated were nifedipine^103^, ^104^ and nicardipine.^100^

###### 3.4.1.2.2 Antihypertensive versus other agents

In RCTs comparing one antihypertensive with another, most participants came from one three‐armed trial of 897 women, comparing oral nifedipine with, uniquely, oral labetalol and oral methyldopa.^88^ Antihypertensives (vs. hydralazine) had no impact on adverse FHR effects (RR 0.71, 95% CI 0.29–1.72, *I* ^2^ = 13%; 3.0% vs. 3.9%; 11 trials, 727 participants), assessed a median of 1.5 [IQR 1–3] hours after drug administration (4 trials^79^, ^81^, ^94^, ^95^) (Figure 3B). The antihypertensives were: labetalol (*N* = 7)^78^, ^81^, ^82^, ^84^, ^87^, ^90^, ^94^ and CCBs (*N* = 5, nifedipine [*N* = 4]^76^, ^79^, ^90^, ^95^ or isradipine^80^). Antihypertensives (vs. CCBs) had no impact on adverse FHR effects (RR 0.52, 95% CI 0.12 to 2.16, I^2^ = 0%; 0.4% vs. 0.7%; 9 trials, 1675 participants), assessed a median of 2 (IQR 1–4) hours after drug administration (Figure 3C); comparisons were of labetalol (*N* = 8 trials,^74^, ^75^, ^77^, ^86^, ^88^, ^90^, ^92^, ^96^ by IV administration in all but one trial^88^ or oral methyldopa^88^), compared with either nifedipine (*N* = 7 trials^74^, ^75^, ^77^, ^88^, ^90^, ^92^, ^96^ or nicardipine^86^). In the Retraction Watch sensitivity analysis excluding Easterling et al.^88^ and Scardo et al.,^75^ the results were unchanged (RR 3.0, 95% CI 0.13–70.83; Figure S3). Two trials reported the pre‐ and post‐ treatment changes in mean FHR between antihypertensive groups.^85^, ^93^ No difference in mean FHR was reported before and after administration of: labetalol and hydralazine,^93^ or labetalol and nifedipine.^85^

In observational studies of one antihypertensive versus another, there was considerable heterogeneity overall (RR 0.34, 95% CI 0.10–1.14, *I* ^2^ = 87%; 11.7% vs. 35.1%; 4 studies, 590 participants), and within the labetalol (vs. hydralazine) subgroup,^98^, ^105^, ^106^ which appeared to be due to a quasi‐randomized trial at high risk of bias^106^ (Figure 3D), while the two other observational studies were of good quality.^98^, ^105^ An additional study of nifedipine (vs. hydralazine) demonstrated fewer adverse FHR outcomes in the nifedipine group (RR 0.09, 95% CI 0.01–0.68, 4.1% vs. 44.0%; 49 pregnancies).^99^

###### 3.4.1.2.3 Network meta‐analysis

In the NMA sensitivity analysis, there were no significant overall differences in adverse FHR effects between antihypertensive agents versus other agents, but 95% credible intervals were very wide (Table S9).

#### 3.4.2 Adverse effects on other fetal surveillance testing

Among included RCTs, 4 (90 participants) reported fetal Doppler findings in addition to FHR effects, but the nature of the reporting permitted only descriptive analysis. Among women treated for non‐severe hypertension, atenolol (vs. pindolol) was associated with an increase in umbilical artery pulsatility index (PI) (mean change of 0.12) versus pindolol (mean change of −0.10) (*p* = 0.008).^39^ Among women treated for severe hypertension, three included RCTs reported umbilical artery waveforms^89^ or both umbilical artery and middle cerebral artery (MCA) PI and resistance index (RI)^85^, ^93^; no significant differences were found between groups in the following comparisons: nifedipine versus placebo^89^; nifedipine versus hydralazine^89^; labetalol versus nifedipine,^85^ and labetalol versus hydralazine.^93^

No observational studies included reported fetal Doppler findings.

No RCTs or observational studies included reported other aspects of fetal monitoring (i.e., amniotic fluid index or deepest vertical pocket of amniotic fluid, or fetal biophysical profile).

#### 3.4.3 Adverse neoHR effects

##### 3.4.3.1 Treatment of non‐severe hypertension

###### 3.4.3.1.1 Antihypertensive versus placebo/no therapy

**FIGURE 4:**
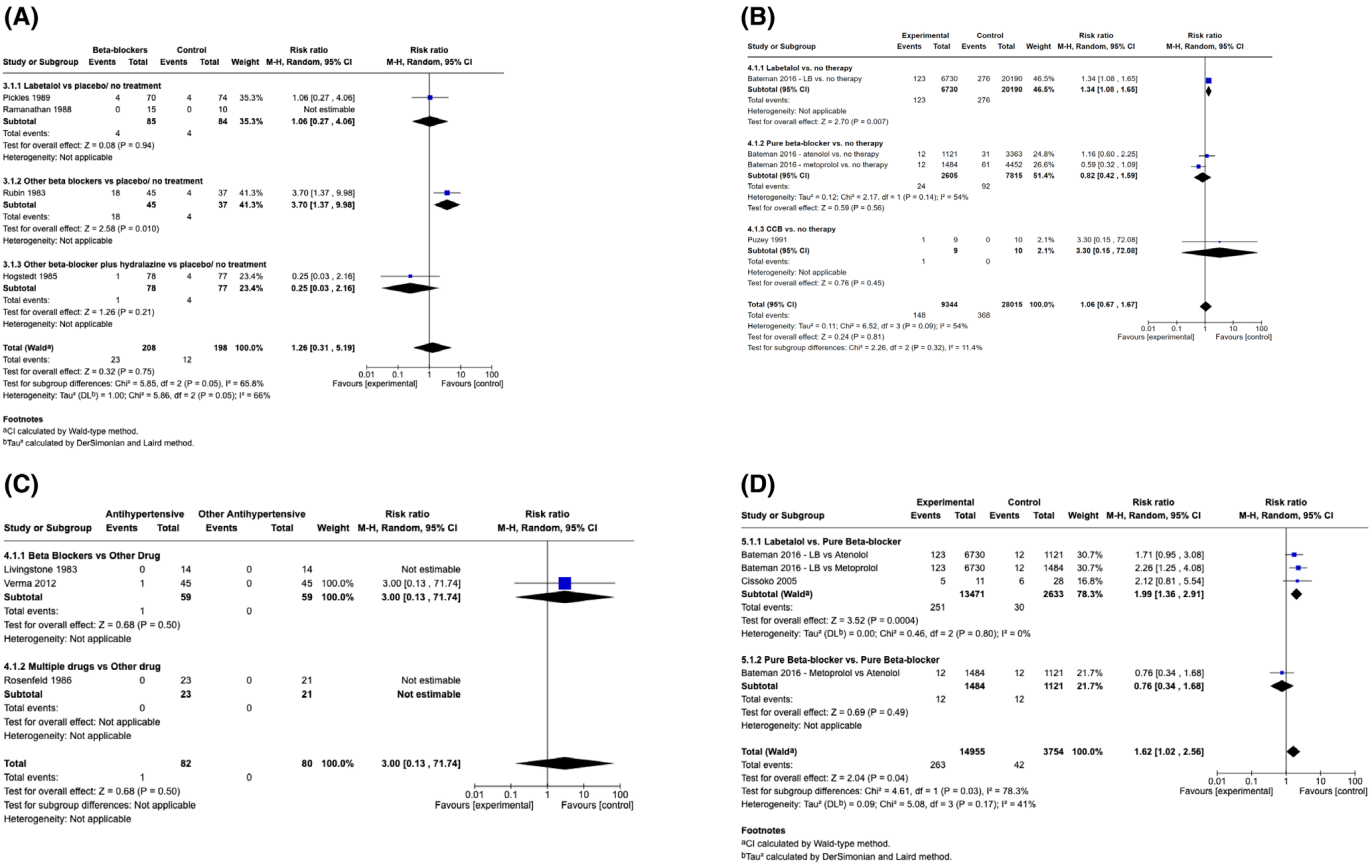
(A) Forest plots of RCTs evaluating the impact of antihypertensives (vs. no therapy) on neonatal HR, among women treated for non‐severe hypertension. (B) Forest plots of OBSERVATIONAL studies showing the effects of antihypertensives (vs. no therapy) on neonatal HR among women with non‐severe hypertension. (C) Forest plots of RCTs showing the effects of antihypertensives (vs. other antihypertensives) on neonatal HR among women with non‐severe hypertension. (D) Forest plots of OBSERVATIONAL studies showing the effects of antihypertensives (vs. other antihypertensives) on neonatal HR among women with nonsevere hypertension. HR, heart rate; RCTs, randomized controlled trials.

In RCTs, antihypertensives (vs. placebo/no therapy) had no impact on neoHR (RR 1.26, 95% CI 0.31–5.19, *I* ^2^ = 66%; 11.1% vs. 6.1%; 4 trials, 406 participants) (Figure 4A). The antihypertensives evaluated were labetalol^35^, ^53^ and pure beta‐blockers (mixed therapy with metoprolol with hydralazine^46^ or atenolol^32^). Substantial heterogeneity appeared to be due to Rubin 1983,^32^ the only trial that undertook continuous neoHR monitoring^32^ and identified no baby who required intervention for neonatal bradycardia.

In observational studies, antihypertensives (vs. no therapy) were not associated with more adverse neoHR effects (RR 1.06, 95% CI 0.67–1.67, *I* ^2^ = 54%; 1.6% vs. 1.3%; 4 comparisons, 37 359 pregnancies) (Figure 4B). Most participants came from the control group of Bateman et al.^66^ The antihypertensives studied were labetalol,^66^ pure beta‐blockers,^66^ or CCBs.^58^ Between‐study heterogeneity appeared to be due to the significant increase in adverse neoHR effects associated with labetalol (vs. no therapy).

###### 3.4.3.1.2 Antihypertensive versus other agents

In RCTs of antihypertensives (vs. other antihypertensives), antihypertensives had no impact on neoHR (RR 3.00, 95% CI 0.13–71.74; 1.2% vs. 0%; 3 trials, 162 participants) (Figure 4C). Only one trial (90 participants), comparing oral/IV labetalol with oral methyldopa reported any neonatal bradycardia event.^52^ One trial (28 participants) compared propranolol with methyldopa^40^ and another(44 participants) compared mixed therapy of pindolol and hydralazine with hydralazine alone.^33^ Two RCTs reported mean neoHR^28^, ^34^; in one trial of methyldopa versus atenolol, “no statistical significance” was found in neoHR between groups,^28^ and in another trial of methyldopa versus labetalol, no difference was found between groups at 1, 24 and 48 h after birth (“*p* > 0.05 for all groups”).^34^

In observational studies, antihypertensives (vs. other antihypertensives) were associated with an increase in adverse neoHR effects when oral labetalol^65^, ^66^ was compared with the oral pure beta‐blockers atenolol or metoprolol (RR 1.99, 95% CI 1.36–2.91, *I* ^2^ = 0%; 1.9% vs. 1.1%; 3 comparisons, 16 104 pregnancies), but not when metoprolol was compared with atenolol (RR 0.76, 95% CI 0.34–1.68; 1 comparison, 2605 pregnancies)^66^ (Figure 4D). It is not known whether the newborns with bradycardia required medical attention.

###### 3.4.3.1.3 Network meta‐analysis

In the NMA sensitivity analysis, there were no observed differences in adverse neoHR effects between antihypertensive agents, including placebo/no therapy, but 95% credible intervals were too wide to draw conclusions (Table S9).

##### 3.4.3.2 Treatment of severe hypertension

No RCTs addressed the impact of antihypertensive therapy on neoHR effects in the setting of severe hypertension.

In a small observational study,^101^ adverse neoHR effects did not differ in labetalol (vs. no therapy) exposed infants (4/55, 7.3% vs. 1/54, 1.9%; *p* = 0.18).^101^ In another small observational study of labetalol versus hydralazine,^102^ no adverse neoHR were reported (0/20 women).

There were no informative data for the NMA sensitivity analysis.

## 4 DISCUSSION

In this systematic review of all available data from randomized and observational studies, antihypertensive agents were administered almost exclusively by the oral route for treatment of non‐severe hypertension, and parenterally (except for nifedipine) for treatment of severe hypertension. In the literature reviewed for treatment of pregnancy hypertension, adverse FHR effects occurred in 7%–9% of pregnancies, and neoHR effects in 1%–6%. Evidence from RCTs is reassuring with regard to antihypertensive effects on FHR or neoHR. However, observational studies (which are larger, more statistically powerful, but of lower quality and with heterogeneous results) raise concerns about a negative impact of beta‐blockers, particularly the alpha/beta‐blocker labetalol, on neoHR, and potentially FHR.

For treatment of non‐severe hypertension, RCT data suggest that there is no clear impact on FHR of antihypertensive therapy (vs. placebo/no therapy), and no clear differential effect of one antihypertensive (vs. another), with the potential of a differential effect of lower doses (≤200 mg/day orally, or 1–4 mg/min intravenously) in more recent studies (potentially no effect of labetalol on FHR) versus a higher dose (≥300 mg/day orally) in the older study (potential adverse effect on FHR; Figure 2A). While the observational data suggest that oral labetalol may be associated with more adverse FHR effects than nifedipine (or Chinese herbal medication), this effect is consistent with the only beta‐blocker versus CCB trial that reported FHR effects (in this case, oral metoprolol vs. oral nicardipine^44^). Of note, there is no evidence that oral methyldopa is inferior to other oral antihypertensives with regard to an impact on FHR, in RCTs or observational studies.

For treatment of severe hypertension in pregnancy, RCTs do not suggest that antihypertensive therapy is associated with adverse FHR effects, with the exception of one small trial that found fewer events for nifedipine (vs. hydralazine). Higher‐quality observational studies are consistent with these findings.

Few studies of antihypertensive therapy for non‐severe hypertension in pregnancy reported neoHR. Some between‐trial differences in effect may have been related to whether neoHR was measured intermittently or continuously, the latter of which would have identified more cases, but not necessarily more cases with clinically important reductions in HR requiring intervention. However, most information was provided by the observational study of Bateman et al.,^66^ which suggested that labetalol, even compared with pure beta‐blockers, is associated with an increase in adverse neoHR effects.

Network meta‐analysis, combining all available literature, failed to reveal any significant associations between antihypertensives and adverse FHR or neoHR effects, and it was not possible to draw reliable conclusions from the very wide 95% CIs. Studies in this update, published from 2001, were either consistent with prior studies or reported on comparisons without prior data.

Other than our previous review of which this is an update, we are unaware of a similar review of the potential effects of antihypertensive on FHR. We have expanded substantially the number of included studies, from 18 trials (1858 women) to 54 (5736 women), and from eight observational studies (139 women) to 28 (2 283 855 women, driven primarily by Bateman et al.'s large record‐linkage study^66^). Nevertheless, our interpretation is similar.^12^ Previously, we observed that there was no evidence of an impact of antihypertensive therapy (for hypertension of any severity) on FHR or neoHR; however, we concluded that the data were inadequate to draw reliable conclusions. With the expanded literature, we note some discordance in results between RCTs (primarily of unclear quality), and observational studies (of variable quality, and showing between‐study heterogeneity in effect estimates) with regards to potential adverse effects of oral labetalol and/or pure beta‐blockers on adverse neoHR effects, and potentially, adverse FHR effects (and potential modification of biological response by ethnicity, as has been raised by others^107^).

Our reported impact on neoHR of “beta‐blockers,” which included labetalol, an alpha‐1 and non‐specific beta‐blocker, relies on the statistical power of the observational study of Bateman et al.^66^ This was a high‐quality data linkage study of 2 292 116 completed pregnancies linked to liveborn infants of Medicaid‐enrolled women (2003–2007), using propensity score‐matching to control for potential confounders of the antihypertensive and neoHR relationship, and undertaking multiple sensitivity analyses. Importantly, two‐thirds of women on “beta‐blockers” were taking labetalol. Not only was neonatal bradycardia associated with neonatal “beta‐blocker” exposure (1.6% in exposed and 0.5% in unexposed), but there was also an association with neonatal hypoglycemia (4.3% in exposed vs. 1.2% the unexposed), strengthening a potential causal relationship. A relationship between beta‐blockers and neonatal hypoglycemia, also reported by Bateman et al., has prompted international pediatric societies to recommend routine monitoring of neonatal capillary blood glucose following maternal beta‐blocker treatment.^108^, ^109^

We are aware of only one review—focused on beta‐blocker therapy (including labetalol)—of the impact on neoHR of antihypertensive therapy to which the fetus is exposed before birth, with the medication to be cleared from their circulation after birth by immature drug clearance mechanisms.^110^ Of 40 studies included in that review (18 trials and 22 observational studies), we excluded five (two RCTs^111^, ^112^) (Table S2) and three observational studies,^113^, ^114^, ^115^ and noted an overlap in two others, which we included as one trial.^32^, ^116^ In addition, we included nine additional studies (five RCTs^28^, ^30^, ^33^, ^34^, ^54^ and four observational studies^59^, ^61^, ^65^, ^102^). Nevertheless, we reached similar conclusions to de Bruin et al.,^110^ raising concern that antenatal labetalol or other pure beta‐blockers may increase the risk of neonatal bradycardia, albeit based on low‐quality evidence. In the de Bruin et al. review, like the study by Bateman et al.,^66^ beta‐blockers were associated with a probable risk of neonatal hypoglycaemia, based on moderate‐quality evidence, but this outcome was not evaluated in our review.

The mechanism of action of labetalol or pure beta‐blockers on the fetal/newborn circulation may be through adrenergic blockade. For labetalol, the most commonly‐prescribed of these medications, animal studies have found inconsistent effects on the uteroplacental circulation or fetal physiology, usually in sheep: a negative impact on uteroplacental perfusion^117^, ^118^ (or not^119^, ^120^); occurrence of fetal lactic acidosis^9^, ^121^; and no adverse effects on FHR or adverse response to acute hypoxemia.^117^

It is not surprising that RCTs have not reported a link between labetalol or pure beta‐blockers and neonatal bradycardia (or hypoglycemia). These are uncommon outcomes, and relevant trials have usually been small and underpowered for effectiveness, and certainly for side effects; the most recent (2018) Cochrane review of antihypertensive therapy during pregnancy did not include FHR as an outcome, and while it did include neonatal bradycardia and hypoglycemia, trials providing data would have been grossly underpowered to find a clinically anticipated effect (i.e., 3 trials, 418 women and 5 trials, 439 women, respectively).^122^ In contrast, large observational studies are better suited to detect safety signals, with the caveat of potential bias associated with such a study design.

Guidelines and educational programs should advise clinicians to ascribe adverse fetal or neonatal heart rate effects to evolving underlying placental dysfunction and not to antihypertensive therapy, based on currently‐available evidence.

Although not considered to be core outcomes in pregnancy hypertension, adverse FHR and neoHR effects should be reported in RCTs and observational studies of antihypertensive therapy, particularly for labetalol and pure beta‐blockers for which there remains some clinical uncertainty.

A strength of our study is the comprehensive inclusion of published literature (randomized and observational), comprehensive quality assessment, and evaluation of both FHR and neoHR effects.

A limitation of our review is that most included trials did not report FHR effects alongside other tests of fetal wellbeing, or describe any modification by existing fetal compromise (e.g., fetal growth restriction). For detection of adverse FHR effects, most studies relied on visual interpretation of the CTG, which is well‐recognized to be unreliable. Other potential confounders of the antihypertensive and outcome relationship were not presented, such as other medications (e.g., antenatal corticosteroids), and we did not have individual‐level data that would enable an analysis of the impact of ethnicity on any FHR or neoHR adverse effects. Most participants initiated antihypertensive therapy in the third trimester of pregnancy, meaning that treatment duration was at most for weeks; however, the elimination half‐life of the most commonly used antihypertensive agents is short (usually <6 h), meaning steady‐state is usually reached within 24 h.^7^

## 5 CONCLUSION

Despite extensive literature review, meta‐ and network meta‐analyses, evidence is inadequate to draw reliable conclusions about the impact of antihypertensives on FHR or neoHR. Based on currently available evidence, it would be prudent to attribute adverse FHR effects to evolving placental dysfunction, and adverse neoHR effects to newborn illness, rather than to maternal antihypertensive therapy.

## AUTHOR CONTRIBUTIONS

AG, primary data collection and review, meta‐analysis (RCT data), manuscript drafting and review. OFB, primary data collection and review, meta‐analysis (observational data), manuscript drafting and review. HDM, study design, data review, manuscript drafting and review. JNB, network meta‐analysis, manuscript drafting and review. MV, study design, manuscript drafting and review. EA, study design, manuscript drafting and review. KB, data review, manuscript drafting and review. AK, study design, manuscript drafting and review. PD, study design, meta‐analysis, manuscript drafting and review. LAM, study design, meta‐analysis, manuscript drafting and review.

## FUNDING INFORMATION

HDM's salary was supported by grants from UK Research and Innovation Grand Challenges Research Fund (MR/P027938/1), the NIHR‐Wellcome Partnership for Global Health Research Award (217 123/Z/19/Z), and the National Institute for Health and Care Research (134293).

## CONFLICT OF INTEREST STATEMENT

The authors report no conflicts of interest.

## Supporting information


Appendix S1.

## Data Availability

Data sharing not applicable to this article as no datasets were generated or analysed during the current study.
