# Supplementary material for: Antihypertensive therapy for pregnancy hypertension and implications for fetal and neonatal heart rate monitoring: A systematic review of randomized trials and observational studies
Source: Acta Obstet Gynecol Scand. 2025 Aug 20;104(10):1822–37. doi: 10.1111/aogs.70019 (PMC12451204; doi:10.1111/aogs.70019)
Supplement: Supplementary file 1 — Appendix S1. [file AOGS-104-1822-s001.docx]

# SUPPLEMENTARY APPENDIX

|  | **Title** | **Page number** |
| --- | --- | --- |
| **TABLES** |  |  |
| S1 | The search strategies used for the electronic database search | 2 |
| S2 | Randomised controlled trials included that did not report outcomes of interest | 3 |
| S3 | Characteristics of the included randomised controlled trials which reported fetal or neonatal heart rate outcomes | 8 |
| S4 | Characteristics of the included observational studies which reported on outcomes of interest | 20 |
| S5 | Risk of bias assessments for the included randomised and quasi-randomised controlled trials | 28 |
| S6 | Risk of bias assessment with the Newcastle Ottawa Scale for controlled observational studies | 31 |
| S7 | Risk of bias assessment with the Johanna Briggs Institute Checklist for the included case series | 32 |
| S8 | Maternal and perinatal outcomes reported in studies of antihypertensive medications that reported outcomes of adverse fetal or neonatal heart rate effects | 33 |
| S9 | Network meta-analysis results for the impact of antihypertensive therapy on fetal or neonatal heart rate | 34 |
| **FIGURES** |  |  |
| S1 | Forest plots of RCTs assessing the impact of antihypertensives (vs. placebo/no therapy) on adverse FHR effects, among women treated for non-severe hypertension – SENSITIVITY ANALYSIS for Figure 1 | 36 |
| S2 | Forest plots of RCTs assessing the impact of labetalol or other beta-blockers (vs. other antihypertensives) on adverse FHR effects, among women treated for non-severe hypertension – SENSITIVITY ANALYSIS for Figure 3 | 37 |
| S3 | Forest plots of RCTs evaluating the impact of antihypertensive therapy (vs. CCBs) on adverse FHR effects, among women with severe hypertension – SENSITIVITY ANALYSIS for Figure 7 | 38 |

#

# Table S1: The search strategies used for the electronic database search

| **Databases** | **Key words** |
| --- | --- |
| **Medline for trials (similar strategy used for PubMed, Embase, CINAHL, CENTRAL, and Web of Science)** | |
|  | {antihypertensive* OR antihypertensive agent* OR methyldopa OR labetalol OR hydralazine OR nifedipine} AND {hypertension OR hypertension[mh] OR hypertensive disorders of pregnancy OR pregnancy-induced hypertension OR preeclampsia OR preeclampsia [mh] OR pregnancy toxemia* OR gestational hypertension OR Hypertension, pregnancy-induced[mh]} AND {Pregnancy[mh] OR Pregnan* OR Gestation* OR pregnant women[mh] OR Pregnancy Complications[mh] OR Postpartum Period [mh] OR Puerperium OR postpartum OR Peripartum Period [mh] OR Peripartum* OR Perinatal Care[mh] OR perinatal} AND { controlled clinical trial [pt] OR clinical trial [pt] OR randomized controlled trials as topic[mh] OR random allocation[mh] OR double-blind method[mh] OR single-blind method[mh] OR “clinical trial”[tw] OR ((single*[tw] OR doubl*[tw] OR trebl*[tw] OR tripl*[tw]) AND (mask*[tw] OR blind*[tw])) OR placebos[tw] OR randomi*[tw] OR research design[mh:noexp] OR comparative study[pt] OR Evaluation Studies[pt] OR Evaluation Study[mh] OR follow-up studies[mh] OR prospective studies[mh] OR control?[tw] OR controll* OR prospective*[tw] OR volunteer*[tw]} |
| **Medline for observational literature (similar strategy used for PubMed, Embase, CINAHL, CENTRAL, and Web of Science)** | |
|  | Labetalol OR Nifedipine OR Methyldopa OR Hydralazine” AND “pregnancy OR hypertension induced pregnancy OR preeclampsia” AND “neonatal OR fetal heart rate OR non-stress test OR cardiotocogram”. |
| **ClinicalTrials.gov (advanced search)** | |
|  | Hypertension, pregnancy induced (in Condition) AND intervention studies |
|  | Preeclampsia (in condition) AND intervention studies |
| **WHO ICTRP** | |
|  | Hypertension AND pregnancy |
|  | Preeclampsia AND pregnancy |
|  | Preeclampsia AND pregnancy |
| **LILACS and Cochrane Pregnancy & Childbirth Trials Register** | |
|  | {hypertension, hypertensive disorders of pregnancy, pregnancy-induced hypertension, preeclampsia, pregnancy toxemias, OR gestational hypertension} AND |
|  | {antihypertensives OR antihypertensive agent} |

CINAHL=Cumulated Index to Nursing and Allied Health Literature; CENTRAL=Cochrane Central Register of Controlled Trials; & WHO=World health organisation; ICTRP=International Clinical Trials Registry Platform; LILACS=Latin America and the Caribbean Literature on

Health Sciences, mh= MeSH.

**Table S2**: RCTs included that did not report outcomes of interest

| **Citation** |
| --- |
| Magee LA, von Dadelszen P, Rey E, Ross S, Asztalos E, Murphy KE, et al. Less-Tight versus Tight Control of Hypertension in Pregnancy. New England Journal of Medicine. 2015;372(5):407-17.  Babbar K, Armo M, Bhanja RL. A comparative study of efficacy of antihypertensive drugs and feto-maternal outcome in the treatment of pregnancy induced hypertension. International Journal of Reproduction, Contraception, Obstetrics and Gynecology. 2015;4(6):1846-53.  Banerjee G, Chatterjee D, Chatterjee A, Mukherjee P, Das A. A randomized controlled trial on use of nimodipine in mild PIH. Journal of Obstetrics and Gynecology of India. 2002;52(4):44-6.  Blake S, Macdonald D. The prevention of the maternal manifestations of preeclampsia bt intensive antihypertensive treatment British Journal of Obstetrics and Gynaecology. 1991;98(3):244-8.  Borghi C, Immordino V, Esposti D. Comparison between nifedipine-GITS and methyldopa on blood pressure control, utero-placental hemodynamic and fetal outcome in patients with pre-eclampsia. Hypertens Pregnancy. 2000;19(8).  Bott-Kanner G, Hirsch M, Friedman S, Boner G, Ovadia J, Merlob P, et al. Antihypertensive therapy in the management of hypertension in pregnancy - a clinical double-blind study of pindolol. Clinical and Experimental Hypertension Part B-Hypertension in Pregnancy. 1992;11(2-3):207-20.  Butters L, Kennedy S, Rubin PC. Atenolol in essential hypertension during pregnancy Bmj-British Medical Journal. 1990;301(6752):587-9.  Magee L, Von Dadelszen P, Chan S, Gafni A, Gruslin A, Helewa M, et al. The control of hypertension in pregnancy study pilot trial. BJOG: An International Journal of Obstetrics & Gynaecology. 2007;114(6):770-e20.  Casavilla F, Ruda Vega H, editors. Prospective and randomized study of mepindolol and alpha-methyldopa efficacy in arterial hypertension (AH) treatment during pregnancy. Proceedings of the World Congress of Gynecology and Obstetrics; 1988.  Catalano D, ES PF, Ascione L, Russo C, De Santi B. Evaluation of nifedipine monotherapy in the management of pregnancy hypertension. Giornale Italiano Di Ostetricia e Ginecologia. 1997;6:373.  Cruickshank DJ, Robertson AA, Campbell DM, Macgillivray I. Maternal obstetric outcome measures in a randomised controlled-study of labetalol in the treatment of hypertension in pregnancy. Clinical and Experimental Hypertension Part B-Hypertension in Pregnancy. 1991;10(3):333-44.  Elhassan EM, Mirghani OA, Habour AB, Adam I. Methyldopa versus no drug treatment in the management of mild pre-eclampsia. East African medical journal. 2002;79(4):172-5.  Eloff W. The use of nifedipine vs methyldopa in mild to moderate pregnancy associated hypertension. Proceedings of the 12th Conf Priorities Perinat Care, South Africa. 1993:130–3.  Faneite PJ, González de Chirivella X, Salazar de Dugarte G. Evaluación de antihipertensivos en embarazadas: mepindolol y alfametildopa. Estudio prospectivo y randomizado. Rev obstet ginecol Venezuela. 1988:139-43.  Freire S, de França L, Rau de Almeida Callou M, Alves de Oliveira J, Barbosa Filho J. Comparative study with pindolol and methyldopa in pregnant women with chronic hypertension. Jornal Brasileiro de Ginecologia. 1988;98(3):157-60.  Gallery EDM, Ross MR, Gyory AZ. Antihypertensive treatment in pregnancy - analysis of different responses to oxyprenolol and methyldopa. Bmj-British Medical Journal. 1985;291(6495):563-6.  Gruppo di Studio Ipertensione G, Parazzini F, Benedetto C, Bortolus R, Ricci E, Marozio L, et al. Nifedipine versus expectant management in mild to moderate hypertension in pregnancy. Britis<[C.Sanchez-Soriano@ed.ac.uk](mailto:C.Sanchez-Soriano@ed.ac.uk)>; ah Journal of Obstetrics and Gynaecology. 1998;105(7):718-22.  Horvath JS, Phippard A, Korda A, Hendersonsmart DJ, Child A, Tiller DJ. Clonidine hydrochloride - a safe and effective antihypertensive agent in pregnancy. Obstetrics and Gynecology. 1985;66(5):634-8.  Kahhale S, Paula F, Sabbaga E, Neme B. Comparative study of chronic hypertensive pregnant women treated and non-treated with pindolol. Ginecologia e ObstetrÃcia Brasileiras. 1985;8:85.  Leather HM, Humphreys DM, Baker P, Chadd MA. A controlled trial of hypotensive agents in hypertension in pregnancy. Lancet. 1968;2(7566):488-+.  Li C, Lao T, Yu K, Wong S, Leung C, editors. The effect of labetalol on mild pre-eclampsia. Proceedings of 7th Word Congress of Hypertension in Pregnancy; 1990.  Mandi, D., Ray, H. C., Shahnaz, I., & Nayek, H. (2023). A Comparative Study of Intravenous Labetalol and Oral Nifedipine for Control of Blood Pressure in Severe Pre-Eclampsia in a Tertiary Care Hospital. *European Journal of Cardiovascular Medicine, 13(2)*, 688-697.  Nascimento D. [Avaliaçäo do uso do Verapamil em gestantes com formas näo graves de doença hipertensiva vascular crônica] Evaluation of the use of Verapamil with non-serious form of chronic vascular hypertension disease during pregnancy 2000.  Nasir, S. A. (2023). Labetalol against Alpha Methyldopa for the treatment gestational hypertention. *Revista Latinoamericana de Hipertension, 18(8)*, 394-399.  Neri I, Valensise H, Facchinetti F, Menghini S, Romanini C, Volpe A. 24-hour ambulatory blood pressure monitoring: A comparison between transdermal glyceryl-trinitrate and oral nifedipine. Hypertension in Pregnancy. 1999;18(1):107-13.  Nimbark, N., Sharma, R., & Jain, S. (2024). Comparison of the Efficacy of Labetalol and Nifedipine in Preeclampsia: A Prospective Interventional Study. *Journal of Clinical and Diagnostic Research, 18(3)*, QC14-QC17.  Odendaal H, Schabort I, Pattinson R. Prazosin for the treatment of hypertension in pregnancy: a randomized control trial. Oxford Database Perinat Trials. 1991.  Paran E, Holzberg G, Mazor M, Zmora E, Insler V. Beta-adrenergic blocking agents in the treatment of pregnancy-induced hypertension. International Journal of Clinical Pharmacology and Therapeutics. 1995;33(2):119-23.  Plouin PF, Breart G, Llado J, Dalle M, Keller ME, Goujon H, et al. A randomized comparison of early with conservative use of antihypertensive drugs in the management of pregnancy-induced hypertension. British Journal of Obstetrics and Gynaecology. 1990;97(2):134-41.  Redman CWG, Beilin LJ, Bonnar J, Ounsted MK. Fetal outcome in trial of antihypertensive treatment in pregnancy. Lancet. 1976;2(7989):753-6.  Sharif N, Usman I, Azhar T. Pregnancy induced hypertension: to compare efficacy of methyldopa and labetalol in management. The Professional Medical Journal. 2016;23(10):1187-93.  Singh, P., Jha, S., & Kumari, U. (2024). Evaluation of the Effectiveness of Intravenous Labetalol and Oral Nifedipine for Managing Severe Hypertension during Pregnancy. *International Journal of Pharmaceutical and Clinical Research, 16(5)*, 1424-1428.  Tuimala R, Hartikainensorri AL. Randomised comparison of atenolol and pindolol for treatment of hypertension in pregnancy. Current Therapeutic Research-Clinical and Experimental. 1988;44(4):579-84.  Vigil-De Gracia P, Dominguez L, Solis A. Management of chronic hypertension during pregnancy with furosemide, amlodipine or aspirin: a pilot clinical trial. Journal of Maternal-Fetal & Neonatal Medicine. 2014;27(13):1291-4.  Voto LS, Zin C, Neira J, Lapidus AM, Margulies M. Kentanserin versus alpha-methyldopa in the treatment of hypertension during pregnancy - A preliminary report. Journal of Cardiovascular Pharmacology. 1987;10:S101-S3.  Voto LS, Lapidus AM, Neira J, Margulies M. Treatment of hypertension during pregnancy: atenolol versus alpha-methyldopa. Obstet y Ginecol Latino-Am. 1985;43:335–41.  Webster LM, Myers JE, Nelson-Piercy C, Harding K, Cruickshank JK, Watt-Coote I, et al. Labetalol Versus Nifedipine as Antihypertensive Treatment for Chronic Hypertension in Pregnancy A Randomized Controlled Trial. Hypertension. 2017;70(5):915-+.  Wichman K, Ryden G, Karlberg BE. A placebo controlled trial of metoprolol in the treatment of hypertension in pregnancy. Scandinavian Journal of Clinical & Laboratory Investigation. 1984;44:90-5.  Malik BA, Momina SB, Ashraf T. Comparison between methyldopa and combination of methyldopa and nifedipine in terms of mean change in blood pressure in pregnancy induced hypertension. Pakistan Journal of Medical Sciences. 2021;15(7):1703-5.  Rauf H, Zubair F, Shaheen S, Laique T. Oral Labetalol Versus Oral Methyldopa for the Management Pregnancy Induced Hypertension. Pakistan Journal of Medical Sciences. 2021;15(4):793-5.  Wang Y, Shi D, Chen L. Lipid profile and cytokines in hypertension of pregnancy: A comparison of preeclampsia therapies. J Clin Hypertens (Greenwich). 2018;20(2):394-9.  Zulfeen M, Tatapudi R, Sowjanya R. IV labetalol and oral nifedipine in acute control of severe hypertension in pregnancy-A randomized controlled trial. Eur J Obstet Gynecol Reprod Biol. 2019;236:46-52.  Bolte AC, van Eyck J, van Schijndel R, van Geijn HP, Dekker GA. The haemodynamic effects of ketanserin versus dihydralazine in severe early-onset hypertension in pregnancy. British Journal of Obstetrics and Gynaecology. 1998;105(7):723-31.  Das S, Biswas S, Das P, Mahapatra B. Comparative study of intravenous labetalol and oral nifedipine for control of blood pressure in severe preeclampsia. J Dent Med Sci. 2015;14:22-7.  De Pasquale SD, Velarde R, Reyes O, De La Ossa K. Hydralazine vs labetalol for the treatment of severe hypertensive disorders of pregnancy. A randomized, controlled trial. Pregnancy Hypertension-an International Journal of Womens Cardiovascular Health. 2014;4(1):19-22.  Howarth GR, Seris A, Venter C, Pattinson RC. A randomized controlled pilot study comparing urapidil to dihydralazine in the management of severe hypertension in pregnancy. Hypertension in Pregnancy. 1997;16(2):213-21.  Jegasothy R, Paranthaman S. Sublingual nifedipine compared with intravenous hydrallazine in the acute treatment of severe hypertension in pregnancy: potential for use in rural practice. The journal of obstetrics and gynaecology research. 1996;22(1):21-4.  Morris R, Sunesara I, Darby M, Novotny S, Kiprono L, Bautista L, et al. Impedance cardiography assessed treatment of acute severe pregnancy hypertension: a randomized trial. Journal of Maternal-Fetal & Neonatal Medicine. 2016;29(2):171-6.  Neto CN, Maia SSB, Katz L, Coutinho IC, Souza AR, Amorim MM. Clonidine versus Captopril for Severe Postpartum Hypertension: A Randomized Controlled Trial. Plos One. 2017;12(1).  Seabe SJ, Moodley J, Becker P. Nifedipine in Acute Hyptertensive Emergencies in Pregnancy South African Medical Journal. 1989;76(6):248-50.  Shekhar S, Sharma C, Thakur S, Verma S. Oral Nifedipine or Intravenous Labetalol for Hypertensive Emergency in Pregnancy A Randomized Controlled Trial. Obstetrics and Gynecology. 2013;122(5):1057-63.  Steyn DW, Odendaal HJ. Dihydralazine or ketanserin for severe hypertension in pregnancy? Preliminary results. European Journal of Obstetrics Gynecology and Reproductive Biology. 1997;75(2):155-9.  Tariq S, Shahid A, Yousof T. Comparison of Maternal Hypotension after administration of Labetalol versus Hydralazine in treating patients having Severe Pregnancy Induced Hypertension. Pakistan Journal of Medical & Health Sciences. 2017;11(2):541-3.  Vigil-De Gracia P, Ruiz E, López JC, de Jaramillo IA, Vega-Maleck JC, Pinzón J. Management of severe hypertension in the postpartum period with intravenous hydralazine or labetalol: a randomized clinical trial. Hypertens Pregnancy. 2007;26(2):163-71.  Walss Rodriguez RJ, Flores Padilla LM. Management of severe pre-eclampsia/eclampsia. Comparison between nifedipine and hydralazine as antihypertensive agents  Manejo de la preeclampsia severa/eclampsia. Comparacion entre Nifedipina e Hidralazina como medicamentos antihipertensivos. Ginecologia y obstetricia de Mexico. 1993;61:76-9.  Sharma C, Soni A, Gupta A, Verma A, Verma S. Hydralazine vs nifedipine for acute hypertensive emergency in pregnancy: a randomized controlled trial. American Journal of Obstetrics and Gynecology. 2017;217(6):687.e1-.e6.  Thakur A, Vashisht R, Agrawal A. Comparison of Intravenous Labetalol with oral Nifedipine for Control of Blood Pressure in Severe Preeclampsia Journal of Hypertension. 2023;41(Suppl 1):e465.  Chaudhari S, Chaudhari D, Arvikar M. A Comparative Study of Labetalol versus Methyldopa in the Treatment of Preeclampsia. International Journal of Pharmaceutical and Clinical Research. 2022;14(5):547-51.  Mehdi MA, Ahmad A, Singh A, Khan S, Singh S, Srivastava K. Comparison of Efficacy of Labetalol with Nifedipine in Patients of Severe Preeclampsia. International Journal of Pharmaceutical and Clinical Research. 2022;14(1):345-55.  Shahi P, Sinha G. A Randomized Comparative Assessment of the Maternal and Fetal Outcomes and Adverse Effects of both Intravenous Labetalol and Oral Nifedipine. International Journal of Pharmaceutical and Clinical Research. 2022;14(7):471-9.  Akhter T, Hashmi KS, Kaynat S, Faridi S. Oral Labetalol in Comparison with Methyldopa in Treatment of Gestational Hypertension as a First Line Drug. Pakistan Journal of Medical & Health Sciences. 2023;17(02):256-. |

RCT (randomised controlled trial)

# Table S3: Characteristics of the included RCTs which reported FHR or neonatal HR outcomes

| Study (Country) | N | Control Arm | Treatment Arm  Details (Drug, dose and route) | | | GA at entry | Severity of HTN | Type of HTN | **Description of adverse FHR/ neonatal HR Effect** | **Delayed FHR/ neonatal HR Outcome** | **Method of Assessing FHR/ neonatal HR** | **Other outcomes** |
| --- | --- | --- | --- | --- | --- | --- | --- | --- | --- | --- | --- | --- |
|  |  |  | 1 | 2 | 3 |  |  |  |  |  |  |  |
| **Non-severe hypertension RCTs** | | | | | | | | | | | | |
| Aparna 2013  (India) | 100 | Other drug | Methyldopa PO 750 - 2000 mg/d | Nifedipine PO 40 - 160 mg/d | NA | T3 | Mild-mod | GH/PET | Abnormal CTG: (a) episode of tachycardia (FHR > 160 bpm) lasting > 5 mins; (b) episode of bradycardia (FHR < 120 bpm) lasting >5 mins; (c) "flattening" of the tracing (lack of variability of FHR to within 5 bpm > 5 mins); or (d) late deceleration (occurring >15 secs from peak of contraction). | 12/45 [Nifed] vs 9/47 [MD] | CTG - NS if computerised | None |
| Arias 1979  (USA) | 58 | No treatment | Mixed Therapy: Methyldopa PO 750–2000 mg/d and Thiazide OR Hydralazine PO 75–250 mg/d and Thiazide OR BOTH | NA | NA | T2 | Mild | Chronic | Antepartum (positive OCT Freeman criteria) or intrapartum evidence (severe variable decelerations, late decelerations of any magnitude, decreased baseline variability) of fetal distress. | 8/29 [Treatment] vs 7/29 [Control] | Visual inspection of CTG | None |
| Digra 2022  (India) | 250 | Other drug | Methyldopa PO 750 - 3000mg/d | Labetalol PO 300 - 800 mg/d | NA | T2 – T3 | Mild-mod | “PIH” | “Lower segment caesarean section due to acute fetal distress” | 9/125 [MD] vs 9/125 [LB] | NS | None |
| El-Guindy 2008  (Egypt) | 120 | Placebo * | Methyldopa PO tight 1076.1+-380.2 mg/d | Methyldopa PO less tight 965.9+-338.9 mg/d | NA | T3 | Mild | Mixed | Non reactive non-stress test | 0/60 [Tight] vs 1/60 [Less tight] | Visual inspection of CTG | None |
| Ellenbogen 1986  (Israel) | 32 | Other drug | Methyldopa PO max 2g/d (14 given hydral) | Pindolol PO 15 mg/d (6 given hydral) | NA | T3 | Mild-mod | “PIH” | Fetal bradycardia and poor reactivity | 0/16 [Pindolol] vs 0/16 [MD] | Visual inspection of CTG | None |
| Fidler 1983  (UK) |  | Other drug | Methyldopa PO 750–3000 mg/d (2 given hydral) | Oxyprenolol PO 160–640 mg/d (6 given hydral) | NA | T3 | Mild | Mixed | Abnormal FHR in labour, (tachycardia > 5 min, bradycardia > 5 min, lack of FHR variability, or late decelerations) | 15/50 [MD] vs. 24/50 [Oxypren] | Visual inspection of CTG | None |
| Högstedt 1985  (Sweden) |  | No treatment | Mixed - Metoprolol PO 50-200 mg/d and Hydralazine PO 25-150 mg/d | NA | NA | T2 - T3 | Mild | GH/PET | Baseline FHR, variability, accelerations of > 15 beats and decellerations. CTG tracings classified into normal, suspicious or abnormal using modified criteria Solum et al. | FHR variability: 23/49 [Treatment] vs 23/57 [Control] | Visual inspection of CTG | None |
|  |  |  |  |  |  |  |  |  | Neonatal bradycardia defined as <120 beats/min | 1/78 [Treatment] vs 4/77 [Control] |  |  |
| Jannet 1994  (France) | 100 | Other drug | Nicardipine PO 80 mg/d | Metoprolol PO 200 mg/d | NA | T2 - T3 | Mild-mod | NS | Fetal distress during labour, defined as FHR decellerations | 3/50 [Nicardipine] vs 14/50 [Metoprolol] | Visual inspection of CTG | None |
| Lamming 1980  (UK) | 26 | Other drug | Methyldopa PO 750-1500 mg/d | Labetalol PO 400-800 mg/d | NA | T3 | Mild | “PIH” | ‘Abnormal’’ FHR tracings, by inspection, during labor | 1/8 [MD] (bradycardia) vs 2/11 [LB] (tachycardia) | Visual inspection of CTG | None |
| Lardoux 1988  (France) | 63 | Other drug | Methyldopa PO 500 - 1500 mg/d | Labetalol PO 400 - 1200 mg/d | Acebutolol PO 400 - 1200 mg/d | T2 | Mild-mod | NS | FHR measurement | “No significant difference” | FHR measurement | None |
| Liu 2022  (China) | 500 | Placebo^#^ | Labetalol IV 1-4mg/min | NA | NA | T3 | Mod | “PIH” | Fetal distress - NS what classifies as this | 9/250 [Placebo] vs 2/250 [LB] | NS | None |
| Livingstone 1983  (Australia) | 28 | Other drug | Methyldopa PO 0.5-1 g/d | Propanolol PO 30-160 mg/d | NA | NS | Mild | “PIH” | Neonatal bradycardia | 0/14 [Propanolol] vs 0/14[MD] | NS | None |
| Montan 1992  (Sweden) | 100 | Other drug | Pindolol PO 5 mg/d | Atenolol PO 50 mg/d | NA | T3 | Mod | Mixed (Gestational, preeclampsia and chronic) | FHR measurement | Significant reduction in FHR observed in Atenolol roup (-11.9 beats/min, p=0.001) | FHR measurement | Fetal Doppler:Increased mean change in umbilical artery PI in atenolol group (0.12 [Atenolol] vs -0.10 [Pindolol] p=0.008) |
| Montan 1996  (Singapore) | 168 | Other drug | Methyldopa PO 500 mg/d | Isradpine PO 5 mg/d | NA | T3 | Mild-mod | Preeclampsia | ‘‘Ominous FHR pattern’’ in labor, by inspection | 1/10 [MD] vs. 1/11 [Isradipine] | Computerised CTG | None |
| Oumachigui 1992  (India) | 30 | Other drug | Methyldopa PO 750 - 2000 mg/d | Metoprolol PO 50 - 300 mg/d | NA | T3 | Mild-mod | “PIH” | FHR measurement | 111+-32 [Metoprolol] 120+-35 [MD]. "There were no adverse effects on … fetal cardiac status" | FHR measurement | None |
| Phippard 1991  (Australia) | 50 | Placebo | Mixed: Hydralazine PO (+clonidine) max 200 mg/d | NA | NA | T1-T2 | Mild-mod | NS | ‘‘Fetal distress on CTG’’ by inspection, weekly | 0/25 [Hydral + clonidine] vs. 0/25 [Placebo] | Visual inspection of CTG | None |
| Pickles 1989  (UK) | 144 | Placebo | Labetalol PO 300-600 mg/d | NA | NA | T2 - T3 | Mild-mod | GH/PET | Neonatal bradycardia defined as <120 bpm | 4/70 [LB] vs 4/74 [Placebo] | NS | None |
| Plouin 1988  (France) | 176 | Other drug | Methyldopa PO 500 - 1500 mg/d | Labetalol PO 400 to 1200 mg/d | NA | T2 - T3 | Mild-mod | Mixed | Mean neonatal heart Rate measured at 1, 12, 24 and 48 hr (bpm) | Mean heart rate did not differ between groups: 1 hr 135 [LB] vs. 137 [MD], 12 hr 129 [LB] vs. 131 [MD], 24 hr 134 [LB] vs. 132 [MD], and 48 hr 133 [LB] vs. 132 [MD] “p>0.05 for all comparisons” | NS | None |
| Ramanathan 1988 (USA) | 25 | No treatment | Labetalol IV 20 mg , followed by 10mg/2 min |  |  | T3 | Mild-mod | Preeclampsia | Undefined Bradycardia, mean neonatal HR | Neonatal bradycardia 0/15 [LB] vs 0/10[no treatment]. Mean HR 138.2 ± 2.5 [LB] VS144 ± 3.2 [no treatment]. |  |  |
| Rosenfeld 1986  (Israel) | 44 | Other drug | Mixed: Pindolol PO 10mg/d and Hydralazine PO 50 mg/d | Hydralazine PO 50 - 2000 mg/d | NA | T2 - T3 | Mild-mod | Mixed (Chronic, GH and PIH) | Neonatal heart rate within normal limits | 0/23 [Pindolol and hydral] vs 0/21 [Hydral] | NS | None |
| Rubin 1983  (UK) | 120 | Placebo | Atenolol PO 100-200 mg/d | NA | NA | T3 | Mild-mod | GH/PET | Neonatal bradycardia, defined as <120 bpm | Bradycardia: 18/45 [Atenolol] vs 4/37 [Placebo] | NS | None |
| Sibai 1987  (USA) | 200 | No treatment | Labetalol PO 300 - 2400 mg/d | NA | NA | T3 | Mild | Preeclampsia | Antepartum 'abnormal FHR testing' as an indication for delivery - NS what classifies as abnormal | 6/94 [No treatment] vs 10/92 [LB] | Visual inspection of CTG | None |
| Sibai 1990  (USA) | 300 | No treatment | Methyldopa PO 750 - 4000 mg/d | Labetalol PO 300 - 2400 mg/d | NA | T1 | Mild | Chronic | Neonatal bradycardia | None in Labetalol exposed | NS | None |
| Sibai 1992  (USA) | 200 | No treatment | Nifedipine PO 40 - 120 mg/d | NA | NA | T3 | Mild | Preeclampsia | Antepartum 'abnormal FHR testing' - NS what classifies as abnormal | 5/99 [No treatment] vs 8/98 [Nifed] | Visual inspection of CTG | None |
| Thorley 1984  (UK) | 60 | Other drug | Methyldopa PO 750 mg/d | Atenolol PO 100 mg/d |  | T2 - T3 | Mild-mod | NS | Mean neonatal heart rate between both groups | "Not statistically significant between both groups" | NS | None |
| Verma 2012 (India) | 90 | Other drug | Methyldopa PO 250mg (max 2000mg/d) | Labetalol oral/IV 100-200mg. Max 1200 mg/d |  | T2-T3 | Mod | NS | Neonatal bradycardia | 1/44 [LB] vs 0/44 [MD] | NS | None |
| Walker 1982  (UK) | 126 | No treatment | Labetalol PO 200 - 1200 mg/d | NA | NA | T2 - T3 | Mild | Mixed | Abnormal antenatal CTG defined as instances of bradycardia, loss of variability or accelerations | 0/64 [LB] vs 0/62 [Control] | Visual inspection of CTG | None |
| Weitz 1987  (USA) | 25 | Placebo | Methydopa PO 750-2000 mg/d | NA | NA | NS | Mild-mod | Chronic | Fetal distress - NS what classifies as this | No difference between groups | Fetal scalp electrode | None |
| Wide-Swensson 1995  (Sweden) | 118 | Placebo | Isradipine PO 10 mg/d | NA | NA | T3 | Mod | NS | Abnormal antenatal CTG | 0/57 [Isradipine] vs 0/57 [Placebo] | Visual inspection of CTG | None |
| Wu 2024 (China) | 90 | No treatment | Labetalol PO 200 mg/d |  |  | T3 | Moderate | NS | “Fetal intrauterine distress” | 0/45 [LB] vs 4/45 [no treatment] | NS | None |
| **Severe hypertension RCTs** | | | | | | | | | | | | |
| Aali 2002  (Iran) | 126 | Other drug | Nifedipine SL 8 mg (max 48mg) | Hydralazine IV 5mg (max 45mg) | NA | T3 | Severe | NS | "Abnormal CTG strip" | 0/65 [Nifed] vs 0/61 [Hydral] | Visual inspection of CTG | None |
| Anilasree 2023 (India) | 106 | Other drug | Nifedipine Oral 10 mg (max 50mg) | Labetalol IV 20mg (max 300mg) |  | T2-T3 | Severe | NS | “Cardiotocographic abnormality” | 0/53 [Nifed] vs 0/53 [LB] | NS | None |
| Ashe 1987  (South Africa) | 20 | Other drug | Labetalol IV 200mg | Dihydralazine IV 25mg | NA | T3 | Severe | NS | One case of late deceleration and two cases of late deceleration during induction of labor | 3/10 [LB] vs 0/10 [Dihydral] | Visual inspection of CTG | None |
| Baggio 2011  (Brazil) | 16 | Other drug | Labetalol IV 20 mg (max 220 mg) | Hydralazine IV 5-10mg every 20 minutes until target BP reached max? | NA | T3 | Severe | NS | FHR before and after treatment “no significant differences were observed” | Before and after (mean FHR) 143.13 ± 6.47 bpm vs. 141.38 ± 10.58 bpm p=0.59 [LB] Before and after (mean FHR) 147.87 ± 9.36 bpm 144.75 ± 9.12 bpm p=0.26 l[Hydral] | NS | Fetal Doppler: No difference in the changes in umbilical artery PI (0.03 [LB] vs 0.05 [Hydral] p=0.87), RI (-0.01 [LB] vs 0.02 [Hydral] p=0.50) and MCA PI (0.15 [LB] vs 0.02 [Hydral] p=0.52) and RI (0.02 [LB] vs -0.02 [Hydral] p=0.33) |
| Baske 2022  (India) | 75 | Other drug | Nifedipine PO 10mg | Labetalol IV 20mg (max 140mg) | NA | T3 | Severe | NS | Incidence of fetal tachycardia | 0/40 [Nifed] vs 0/35 [LB] | NS | None |
| Cleary 2023  (USA) | 110 | Placebo | Extended release nifedipine PO 30mg | NA | NA | T3 | Severe | Preeclampsia | Non reassuring fetal status as an indication for delivery | 3/53 [Nifed] vs 7/49 [Placebo[] | NS | None |
| Donel 2022  (Indonesia) | 49 | Placebo^**^ | Nifedipine PO 20mg (max 60mg) | Labetalol IV 20mg (max 60mg) | NA | T3 | Severe | NS | FHR abnormality | 0/17 [Nifed] vs 0/13 [LB] vs 0/19 [Hydral] | NS | None |
| Duggan 1992 (New Zealand) | 15 | Other drug | Nifedipine PO 10mg | Hydralazine IV 10mg | NA | > 28 Weeks | DBP 105–109 mmHg | NS | CTG scores | Not different between groups | Visual inspection of CTG and Umbilical artery velocimetry | Fetal Doppler. Nifedipine vs placebo: Umbilical artery PI changes were not significantly different between groups (p=0.7) and PI levels were not significantly different between groups (p = 0.7). Nifedipine vs hydralazine: No significant trend occurred with umbilical artery waveforms (p = 0.9) |
| Easterling 2019  (India) | 894 | Other drug | Nifedipine PO 10 mg (max 30mg) | Labetalol PO 200mg (max 600mg) | Methyldopa oral | T3 | Severe | NS | Caesarean section for fetal distress during study period or up to 2 hours after end of study period | 3/298 [Nifed] vs 1/295 [LB] vs 1/301 [MD] | NS | None |
| Ehikioya 2022  (Nigeria) | 120 | Other drug | Hydralazine IV 5mg (max 25mg) | Labetalol IV 25mg (max 300mg) | NA | T3 | Severe | NS | Persistent fetal tachycardia as an indication for caesarean | 4/60 [Hydral] vs 2/60 [LB] | NS | None |
| Elatrous 2002  (Tunisia) | 60 | Other drug | Labetalol IV 1mg/kg (max 2.5 mg/kg) | Nicardipine IV 10mg (max 37.5mg) | NA | T3 | Severe | Mixed (pre-eclampsia and chronic) | FHR abnormalities recorded using external CTG. The one event is "one episode of slight and transient deceleration). | 1/30 [LB] vs 0/30 [Nicardipine] | NS | None |
| Gainder 2019  (India) | 30 | Other drug | Labetalol IV 20mg (max 220mg) | Nifedipine PO 10mg (max 30mg) | NA | T3 | Severe | NS | Changes before and after treatment – “no significant differences”. | Pre treatment: 32.13 ± 6.94 bpm.  Post treatment: 131.47 ± 7.07 bpm p=0.096 [LB] Pre-treatment: 28 + 7.74 bpm   Post treatment: 126.80 + 6.48 bpm p=0.26 [Nifed] | NS | Fetal Doppler: No difference in percentage changes of umbilical artery PI (2.73 [LB] vs -19.9 [Nifed] p=0.052), RI (-4.6 [LB] vs -11.8 [Nifed] p=0.546) and MCA PI (-12.02 [LB] vs -13.03 [Nifed] p=0.927) and RI (-8.37 [LB] vs -20.38 [Nifed] p=0.790) between groups |
| Garden 1982  (South Africa) | 6 | Other drug | Labetalol IV 20 mg/h (max 160 mg/h) | Dihydralazine IV 10mg/h (max 80mg/h) | NA | T3 | Severe | NS | Antepartum fetal distress - abnormal fetal CTG with severe late decelerations. Some degree of bradycardia at birth. Severe bradycardia requiring intervention. | Fetal distress: 0/3 [LB] vs 1/3 [Dihydral]. Fetal bradycardia: 3/3 [LB] vs 0/3 [Dihydral]. Severe fetal bradycardia 1/3 [LB] vs 0/3 [Dihydral]. For purposes of Revman, we have decided to combine fetal distress and severe fetal bradycardia as an outcome (1/3 vs 1/3) | NS | None |
| Gu 2023 (China) | 100 | No Drug | Labetalol IV labetalol 300 mg followed by daily oral 300mg (+MgSO4) | MgSO4 only, therefore analysed as no antihypertensive drug) |  | T3 | Severe | GH | “Fetal intrauterine distress” | 2/50 [LB] VS 4/50 [No treatment] | NS | None |
| Ismail 1993  (Egypt) | 30 | Placebo | Nifedipine PO 60mg/d | NA | NA | T3 | Mod (overall) | Preeclampsia | ‘FHR effects’’ by inspection, before and 30 min, and 3 d and 7 d after nifed/placebo | Acute: 0/20 [Nifed] vs. 0/10 [Placebo] Delayed: 0/20 [Nifed] vs. 0/10 [Placebo] | Visual inspection of CTG | None |
| Khan 2017  (Pakistan) | 78 | Other drug | Labetalol IV 20mg (max 140mg) | Hydralazine IV 5mg (max 15mg) | NA | T3 | Severe | NS | Adverse FHR recordings - "Not noted significantly in either group" | 0/39 [LB] vs 0/39 [Hydral] | NS | None |
| Mabie 1987  (USA) | 19 | Other drug | Labetalol IV 20mg (max 80mg) | Hydralazine IV 5mg repeated based on BP | NA | T3 | Severe | Mixed (pre-eclampsia and chronic) | Abnormal FHR during labor. Two cases were late decelerations | 0/13 [LB] vs 2/6 [Hydral] | NS | None |
| Maharaj 1997  (South Africa) | 40 | Other drug | Isradipine IV 0.15 μg/kg/min increased by 0.0025 μg/kg/min every 15 min based on BP | Dihydralazine IV 6.25mg repeated based on BP | NANA | T3 | Severe | GH/PET | FHR Decelerations | 2/20 [Isradipine] vs 5/20 [Dihydral] | CTG | None |
| Martins Costa 1992  (Brazil) | 37 | Other drug | Nifedipine PO 10-20mg | Hydralazine IV 5-10mg | NA | > 24 Weeks | Severe | Preclampsia | Abnormal FHR pattern (‘‘assessment of baseline and heart rate variability’’), by inspection, for 20 min pre- and 60 min after medication given | 0/20 [Nifed] vs 0/17 [Hydral] | Visual inspection of CTG | None |
| Muhammad 2022  (Nigeria) | 113 | Other drug | Hydralazine IV 10mg (max 50mg) | Labetalol IV 20mg (max 300mg) | NA | T3 | Severe | Mixed (Gestational, Preeclampsia and chronic) | maternal hypotension necessitating urgent delivery due to fetal distress | 1/56 [Hydral] vs 0/57 [LB] | NS | None |
| Raheem 2012  (Malaysia) | 50 | Other drug | Nifedipine PO 10mg (max 50mg) | Labetalol IV 20mg (max 300mg) | NA | T3 | Severe | NS | Significant cardiotoographic abnormalities | 0/25 [Nifed] vs 0/25 [LB] | NS | None |
| Sabir 2016  (Pakistan) | 100 | Other drug | Nifedipine PO 10mg (max 50mg) | Hydralazine IV 5mg (max 25mg) | NA | T3 | Severe | NS | Bradycardia >90 bpm, adverse FHR was recorded at 30 minutes in hydralazine group and 45 minutes in Nifedipine group | 3/50 [Nifed] vs 1/50 [Hydral] | NS | None |
| Scardo 1999  (USA) | 12 | Other drug | Nifedipine PO 10mg (max 30mg) | Labetalol IV 20mg (max 300mg) | NA | T3 | Severe | NS | "No instances of fetal late decellerations" | 0/6 [Nifed] vs 0/6 [Hydral] | NS | None |
| Shi 2016  (China) | 147 | Other drug | Labetalol UV 20mg (max 140mg) | Nifedipine PO 10mg (max 50mg) | NA | T3 | Severe | NS | Cardiotocographic abnormalities | 0/74 [Nifed] vs 0/73 [LB] | NS | None |

bpm=beats per minute; CTG=cardiotocography; IV=intravenous; d=day; mod=moderate; Dihydral=Dihydralazine;. Hydral=Hydralazine; LB=Labetalol; MCA=Middle cerebral artery; Mod=moderate; NA= Not applicable, NS=Not specified; PI= Nifed=Nifedipin; Pulsatility index; “PIH” (pregnancy-induced hypertension, as used by the authors); PO=by mouth; RI=Resistance index; T1=first trimester; T2=second; trimester; T3=third trimester.

*"Less tight" control is analysed as placebo in this study.

^#^Both arms received MgSO4 so this trial is was analysed as LB vs placebo.

^**^Both arms received Nifedipine and MgSO4, so this trial was analysed as Labetalol vs placebo.

Table S4: Characteristics of the included observational studies which reported on outcomes of interest

| **Study**  **(Country)** | **Study Participant (Route of Administration)** | **N women** | **Control Arm** | **Treatment Arm** | **GA at entry** | **Severity of HTN**  **(Type of HTN)** | **Description of adverse FHR/ neonatal HR Effect** | **Delayed FHR/ neonatal HR Outcome** | **Method of Assessing FHR/ neonatal HR** | **Other outcomes** |
| --- | --- | --- | --- | --- | --- | --- | --- | --- | --- | --- |
|  |  |  | Placebo/no treatment/ drug |  |  |  |  |  |  |  |
| Al Ismaili  2022 (Oman) | Fetal  (Oral) | 244 | LAB | Methyldopa | T2-T3 | Non-severe  (Mixed) | Fetal bradycardia undefined, but not in labour. | 8/170 VS 1/74 | Visual inspection of CTG | Preterm birth, SGA, NICU admission, neonatal seizures. |
| Bateman 2016 (USA) | Neonatal  (Oral) | 34,370 | LAB | 1 Atenolol  2. Metoprolol | T3 | Non-Severe  (Mixed) | The presence of neonatal bradycardia was defined as documentation of bradycardia in the new-born by a clinician in the medical record, HR of ≤100, and/or treatment with rate control medications.” | 11,659/ 2,281,531 vs 165/10,585 | Neonatal echocardiograph | None |
| Boutroy 1982 (France) | Neonatal  (Oral) | 31 | Acebutolol | No Control Group. | T3 | Non-severe | Basal HR < 120 beats per minute and lasting longer than 1 h | 12/31 (38.7%) | Continuous instantaneous monitoring. | SGA. |
| Cissoko 2005 (France) | Neonatal  (Oral) | 45 | LAB 220mg (100–400) | Other BB (including propranolol, atenolol, acebutolol, bisoprolol, sotalol, celiprolol, betaxolol, metoprolol, nadolol) | T3 | Non-Severe  (Mixed) | “Bradycardia was defined by a frequency HR< 100/min”. | LAB n/N vs Other BBs n/N 5/11 vs 6/11 | NS | None |
| Chera-Aree 2020 (Thailand) | Fetal  (Parenteral) | 49 | LAB 20-220mg | Hydralazine 5-40mg | T3 | Severe  (Mixed) | “Non-reassuring FHR patterns were defined by National Institute of Child Health and Human Development category II or III”. | LAB n/N vs Hydralazine n/N 21/64 vs 32/62 | Visual inspection of CTG | Placental abruption,  pulmonary edema, raised liver enzymes, newborn death. |
| Dumez 1981 (France) | Neonatal  (Oral) | 20 | Acebutolol | Methyldopa | T3 | Non-severe  (NS) | Abnormal HR undefined. | HR was significantly lower during the first  three days of life in the infants of mothers given acebutolol | Daily during the 3 first days after birth, when the babies were sleeping. | None |
| El-Qarmalawi 1994 (Kuwait) | Fetal  (Oral) | 104 | LAB 100 mg tds | Methyldopa 250 mg tds | T3 | Non-Severe  (GH) | Abnormal FHR undefined | "As regards the fetal/neonatal outcome, there was no significant difference between the two groups. FH changes during labor were similar." LAB 15/54 vs Methyldopa 12/50 | Visual inspection of CTG | Preterm birth. |
| Fenakel 1991 (Israel) | Fetal  (Oral) | 49 | NIF 10-30mg, then PO 40-120 mg/d | Hydralazine IV 6.25-12.5 mg, then PO 80-120 mg/d | T3 | Severe  (Pre-eclampsia) | Acute fetal distress | 1/24 [NIF] vs 11/25 [Hydral] | NS | Preterm birth, neonatal death. |
| Giannubilo 2012 (Italy) | Fetal  (Oral) | 242 (190 non-severe hypertension and 52 severe hypertension) | LAB 100mg | NIF 20mg | T2 | Mixed  (Mixed) | “Abnormal FHR racing was used to assess fetal worsening.” However, abnormal FHR tracing was undefined. | LAB n/N vs NIF n/N 17/64 vs 43/178 | Visual inspection of CTG | HELLP,  NICU admission. |
| Hanff 2005 (Netherlands) | Fetal  (Parenteral) | 27 (studied pre- and post- medication) | Standard therapy  (hypertensive pregnancy), which was methyldopa & ketanserin  Pre-post delivery | Nicardipine 3mg/hour | Mixed | Severe  (Mixed) | Appearance of decelerations and effect on baseline FHR | “No changes in the baseline FHR were observed.” | Visual inspection of CTG | Stillbirth, preterm birth, HELLP, NICU admission, neonatal respiratory support. |
| Heida 2011 (Netherlands) | Neonatal  (Parenteral) | 109 | No therapy  (hypertensive pregnancy) | LAB IV bolus 20mg, then continuous 20mg/hour | T3 | Severe  (Pre-eclampsia) | Bradycardia in the first minutes after birth and during the first 48 h (defined as HR < 100 bpm) | LAB n/N vs no therapy n/N 4/55 vs 1/54 | NS | Newborn death, SGA |
| Hjertberg 1988-1989 (Sweden) | Neonatal  (Mixed) | 20 | Oral Hydralazine 25 mg or IV 1.5 mg | Oral LAB 300 mg or 25 mg IV | T3 | Severe  (Pre-eclampsia) | Abnormal FHR undefined | Mean arterial pressure, HR and respiratory rate did not differ between the groups 0/11 Hydralazine vs 0/9 LAB | A non-invasive oscillo- metric method (Dynamap) | Newborn death, SGA. |
| Li 2021 (China) | Fetal  (Parenteral) | 20 | LAB 50 mg | Chinese Herb | Mixed | Non-severe  (GH) | Abnormal FHR undefined | LAB 15/63 vs Chinese Herb 3/63 | Visual inspection of CTG | Postpartum hemorrhage. |
| Lindow 1988 (South Africa) | Fetal  (Oral) | 18 | No therapy  (hypertensive pregnancy) | NIF | T3 | Severe  (Mixed) | Abnormal FHR undefined | "There was no abnormality in any of the FHR recordings throughout the study" NIF 0/9 vs Placebo 0/9 | Visual CTG | None |
| Lurie 1990 (Israel) | Fetal  (Oral) | 102 | No therapy  (hypertensive pregnancy)  Pre-post design | NIF 10mg | T3 | Severe  (GH) | Abnormal FHR undefined | “No FHR abnormalities were observed in all 51 cases” | Visual CTG | None |
| Molvi 2012 (India) | Fetal  Oral | 149 | Another drug | 1.Methyldopa PO 500 - 2000 mg/d  2.LAB PO 200 - 2400 mg/d | T2-T3 | Non-severe (GH) | Abnormal FHR undefined | 0/50 [Standard care] 0/50 [LB] 0/49 [MD] | NS | Maternal death |
| Montan 1993 (Singapore) | Fetal  (Oral) | 20 (studied pre- and post-medication) | No therapy  (hypertensive pregnancy)  Pre-post study design | Methyldopa 250mg | T3 | Non-Severe  (GH) | Abnormal FHR undefined | FHR remained unchanged during methyldopa treatment”. Mean and SD before treatment: 142.0 ± 9.1 Mean and SD during treatment 143.9 ± 9.6 Mean difference 1.9 ± 11.1 | Visual inspection of CTG | None |
| Munshi 1992 (India) | Neonatal  (Oral) | 129 | LAB | Other antihypertensives | T3 | Non severe  (GH) | HR < 100bpm | 6/48 (12.5%) vs 4/81 (5%) | NS | SGA |
| O’Hare  1980 | Neonatal.  (Oral) | 12 | Sotalolol | No control group | T3 | Non-severe | HR < 120bpm | 6/12 (50%) | Continuous HR monitoring | Hypoglycaemia.  Neonatal death. |
| Pirhonen 1990 (Finland) | Fetal  (Oral) | 12 (studied pre- and post- medication) | No therapy  (hypertensive pregnancy)  Pre-post study design | NIF 20 mg | T3 | Non-Severe  (GH) | Abnormal FHR undefined | “The FHR was 130 ± 6 bpm before treatment and 136 ± 7 bpm at time of second doppler measurement. No significant changes were observed after NIF intake” | Visual inspection of CTG | None |
| Puzey 1991 (South Africa) | Fetal  (Oral) | 20 | Placebo containing vitamin syrup | NIF 5 mg | T3 | Non-Severe  (NS) | Abnormal FHR defined as <120bpm or >160bpm | Mann-Whitney non-parametric V-test showed no significant change in the FHR. NIF 1/9 vs Placebo 0/10 | Visual inspection of CTG | None |
| Richter 2016 (Netherlands) | Neonatal  (Parenteral) | 11 | LAB | NIF | Mixed | Non-Severe  (Mixed) | Abnormal HR undefined | HR in first 48 hours (as median [IQR]): LAB±MgSO4 (N=11) 149 [137-155], NIF±MgSO4 (N=7) 154 [150-161], MgSO4 (N=148) 148 [139-156] | NIRS | Raised liver enzymes, preterm birth. |
| Sandstrom  1982  (Sweden) | Fetal  (Oral) | 184 | metoprolol + hydralazine | Bendroflumethiazide + metoprolol or Bendroflumethiazide + hydralazine | Mixed | Non-Severe | HR <100bpm | 7/101 vs. 1/83  16/97 vs. 1/83 | NS | Perinatal death, IUGR |
| Sharma 2016 (USA) | Fetal  (Parenteral) | 100 | LAB | Hydralazine | Mixed | Severe  (NS) | “The National Institute for Child Health and Human Development (NICHD) three-tier category system was used to describe the FHT (10). Because the NICHD category II includes such a broad range of FHT Abnormalities, Parer and Ikeda’s classification scheme (namely blue, yellow, and orange) were used to further characterize all category II tracings” | LAB 3/25 vs Hydralazine 10/57 | Visual inspection of CTG | None |
| Thewissen 2016 (Netherlands) | Neonatal  (Oral) | 44 | No treatment  (hypertensive pregnancy) | LAB 480 [100– 2400] mg/24h | Mixed | Non-Severe  (Mixed) | Abnormal HR undefined | HR stated to be not significantly different between groups during 4 periods and always between normal clinical limits (mean ± sd 147 ± 14.6 beats per minute (bpm)). HR and LAB dose were not significantly correlated”. Results were presented only as box plots. | NS | Neonatal respiratory support, SGA |
| Verhagen 2016 (Netherlands) | Neonatal  (Oral) | 49 | LAB | NIF | Mixed | Non-Severe  (NS) | Abnormal FHR undefined | Only preterm newborns. HR in first 24 hours. Median [IQR]: LAB±MgSO4 (N=9) 143 [140-155], LAB±MgSO4 + NIF (N=3) 141 [135-145], NIF (N=19) 149 [137-159], No antihypertensive (N=18) 149 [138-165] | NIRS | Raised liver enzymes, preterm birth. |
| William 1982 (United Kingdom) | Neonatal  (Oral) | 20 | Acebutolol | Methyldopa | Mixed | Non-severe | Abnormal FHR undefined | 0/9 vs 0/11 | NS | Hypoglycemia. |
| Yousaf 2023 | Fetal  (Parenteral) | 360 | Hydralazine | LAB | Mixed | Severe | Aberrant FHR (defined as a FHR differing from 100-160 beats per minute within 60 minutes after drug administration). | A total of 55/360 women (16.68%) developed abnormal FHR, with 1 (0.61%) belonging to the LAB group, whilst in the Hydralazine category, FHR accounted for 54 (32.72%) of the total. | NS | None |

BB= Beta blockers, bpm= beats per minute CTG= Cardiotocogram, FHR: fetal heart rate, FH= Fetal heart, FHT= Fetal Heart Tracing, GH= Gestational Hypertension, HDP= Hypertensive disorder of pregnancy, HELLP= Hemolysis Elevated Liver Enzyme and Platelet syndrome, HR=heart rate, IQR=interquartile range, LAB= Labetalol, mg= milligram, MgSO4=magnesium sulphate, NICHD= National Institute of Child Health and Human Development, NICU= Neonatal Intensive Care Unit, NIF=nifedipine, NIRS= Near infrared spectroscopy, NS=Not specified ; SGA= Small for Gestational Age, T1=first trimester; T2=second trimester; T3=third trimester, VS= versus.

Table S5: Risk of bias assessments for the included RCTs and quasi-RCTs

|  | Random generation | Concealment | Blinding | Blinding outcome assessment | Incomplete data (LTFU) | Selective reporting | Overall RoB |
| --- | --- | --- | --- | --- | --- | --- | --- |
| **RCTS of non-severe hypertension** | | | | | | | |
| Aparna 2013 | Series of random numbers | Not described | Open label | No | 8% | Published report only | B |
| Arias 1979 | Not described | Not described | Open label | No | No | Published report only | B |
| Digra 2023 | Computer Generated | Not Described | Open Label | No | No | Published report only | B |
| El-Guindy 2008 | Computer generated | Sealed opaque envelopes | Double blind | yes | 4% | Published report only | A |
| Ellenbogen 1986 | Not described | Not described | Open label | No | No | Published report only | B |
| Fidler 1983 | Not described | Not described | Open label | No | 4% | Published report only | B |
| Högstedt 1985 | Not described | Envelopes (no details) | Open label | No | 4% | Published report only | B |
| Jannet 1994 | Computer generated | Sealed envelopes | Open label | No | No | Published report only | B |
| Lamming 1980 | Random number table | Not described | Open label | No | No | Published report only | B |
| Lardoux 1988 | Not described | Not described | Open label | No | No | Published report only | B |
| Liu 2022 | Not described | No Described | Open Label | No | No | Published report only | B |
| Livingstone 1983 | Not described | Not described | Open label | No | No | Published report only | B |
| Montan 1992 | Not described | Sealed envelopes | Double blind | yes | 9.4% | Published report only | B |
| Montan 1996 | Not described | Sealed envelopes | Open label | No | 22% | Published report only | B |
| Oumachigui 1992 | Not described | Not described | Open label | No | No | Published report only | B |
| Phippard 1991 | Numerical randomisation code | Adequate | Double blind | Yes | No | Published report only | A |
| Pickles 1989 | Random number table | Consecutively-numbered treatment boxes | Double blind | Yes | 5.3% | Published report only | A |
| Plouin 1988 | Block randomisation | Sealed envelopes | Open label | No | 6.4% | Published report only | B |
| Ramanathan 1988 | Not described | Not described | Open label | No | No | Published report only | B |
| Rosenfeld 1986 | Not described | Not described | Open label | No | No | Published report only | B |
| Rubin 1983 | Not described | Not described | Double blind | Yes | No | Published report only | B |
| Sibai 1987 | Computer generated | Sealed envelopes | Open label | No | 0.0 | Published report only | B |
| Sibai 1990 | Computer generated | Sealed envelopes | Open label | No | 12% | Published & unpublished | B |
| Sibai 1992 | Computer generated | Sealed envelopes | Open label | No | 1.5% | Published report only | B |
| Thorley 1984 | Not described | Not described | Open label | No | 4% | Published report only | B |
| Verma 2012 | Computer generated | Unclear | Open label | No | No | Published report only | B |
| Wide-Swensson 1995 | Block randomisation | Not described | Double blind | Yes | 6% | Published report only | B |
| Walker 1982 | Not described | Envelopes (no details) | Open label | No | No | Published & unpublished | B |
| Weitz 1987 | Not described | Not described | Double blind | Yes | No | Published report only | B |
| Wu 2024 | Digital random number table | Not described | Open labetl | Unclear | No | Published report Only | B |
| **RCTS of severe hypertension** | | | | | | | |
| Aali 2002 | Block randomisation | Sealed, opaque envelopes | Single blind | Yes | None | Published &unpublished | B |
| Anilasree 2023 | Computer generated | Non described | Double blind | Unclear | Non | Published Only | B |
| Ashe 1987 | Not described | Not described | Unclear | Unclear | None | Published only | B |
| Baggio 2011 | Not described | Not described | Unclear | Unclear | 5.90% | Published data only | B |
| Baske 2022 | Not Described | Envelopes | Double Blind | Yes | None | Published only | B |
| Cleary 2023 | Block randomisation | Pharmacy | Double Blind | Yes | None | Published only | A |
| Donel 2022 | Computer generated | Not described | Double Blind | Unclear | 18.03% | Published only | B |
| Duggan 1992 | Not described | Not described | Double blind | Yes | None | Published only | B |
| Easterling 2019 | Computer generated | Sealed, opaque envelopes | Open label | No | 3.60% | Published only | B |
| Ehikioya 2023 | Computer generated | Opaque envelopes | Open label | No | None | Published Only | B |
| Elatrous 2002 | Computer generated | Sealed, opaque envelopes | Open label | No | None | Published only | B |
| Gainder 2019 | Random number table | Not described | Open label | Yes | None | Published only | B |
| Garden 1982 | Not described | Not described | Open label | Unclear | None | Published only | B |
| Gu 2023 | Not described | Not described | Open label | Unclear | None | Published only | B |
| Ismail 1993 | Not described | Not described | Double blind | Yes | None | Published only | B |
| Khan 2017 | Not described | Sealed, opaque envelopes | Unclear | Unclear | None | Published only | B |
| Mabie 1987 | Random number table | Not described | Open label | No | None | Published only | B |
| Maharaj 1997 | Computer generated | Not described | Open label | No | 2.50% | Published only | B |
| Martins Costa 1992 | Envelope selection from jumbled box | Sealed envelopes | Double blind | Yes | None | Published only | A |
| Muhammad 2022 | Computer generated | Medication dispensed by pharmacist | Double Blind | Unclear | 5.84% | Published Only | B |
| Raheem 2012 | Computer generated | Sealed envelopes | Double blind | No | 2.00% | Published only | B |
| Sabir 2016 | Not described | Not described | Unclear | Unclear | Unclear | Published &unpublished | B |
| Scardo 1999 | Computer generated | Medication dispensed by pharmacist | Double blind | Yes | None | Published only | A |
| Shi 2016 | Block randomisation | Medication dispensed by pharmacist | Double blind | Yes | None | Published only | A |
| **Quasi-RCTS** | | | | | | | |
| El-Qarmalawi 1994 | Not described | Not described | Unclear | Unclear | 13.33% | Published only | B |
| Fenakel 1991 | According to week of month | Clearly inadequate- knew allocation by knowing week of month | Open label | No | 9.30% | Published only | C |
| Hjertberg 1993 | Not described | Not described | Unclear | Unclear | None | Published only | B |
| Lindow 1988 | Randomised according to "last digit of folder number" | Not described | Unclear | Unclear | None | Published only | C |
| Molvi 2012 | Manually mixed up | Sealed envelopes | Open label | No | 0.6% | Published report only | **C** |
| Puzey 1991 | Randomised according to "last digit of hospital number" | Not described | Double-blind | Yes | None | Published only | C |
| Yousaf 2023 | Lottery Approach | Unclear | Unclear | No | None | Published report only | **C** |

A=Low risk of bias; B=Risk of bias unclear; C=High risk of bias, LTFU=Loss to follow up.

# Table S6: Risk of bias assessment with the Newcastle Ottawa Scale for controlled observational studies*

| Study ID | Selection | Comparability | Exposure/Outcome |
| --- | --- | --- | --- |
| Al Ismail 2021 | 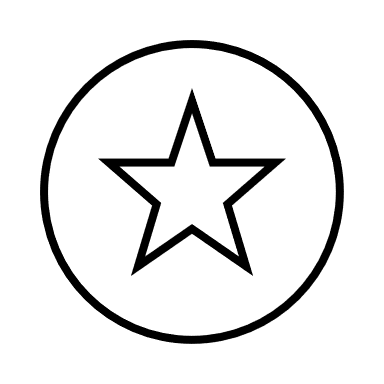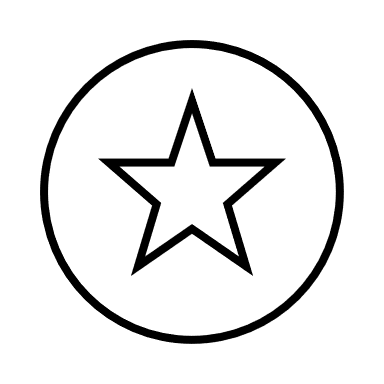 | 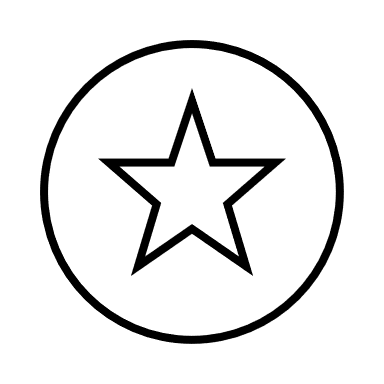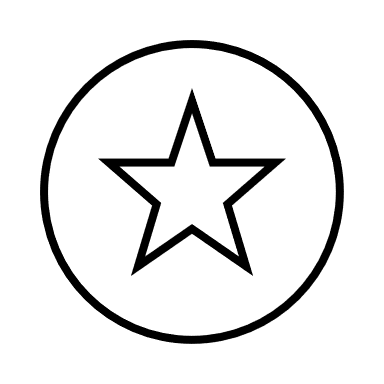 | 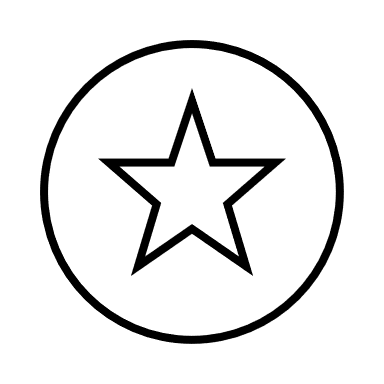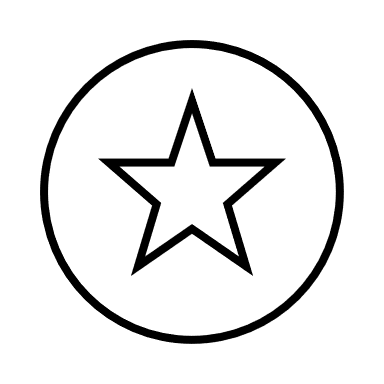 |
| Bateman, 2016 | 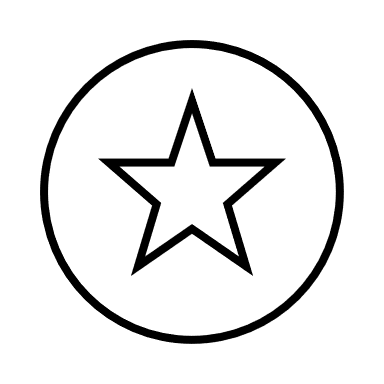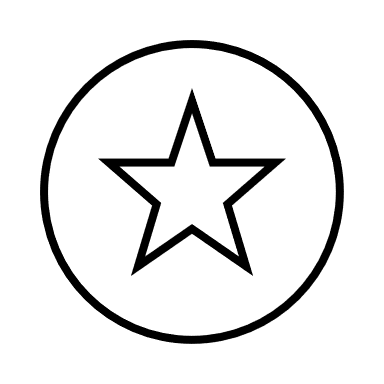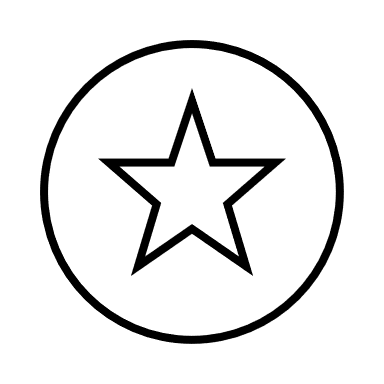 | 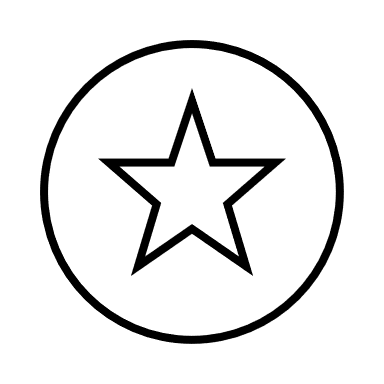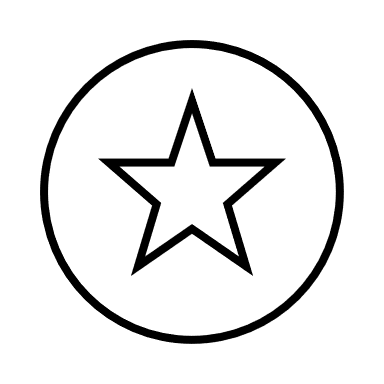 | 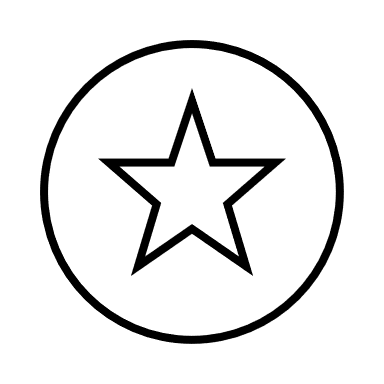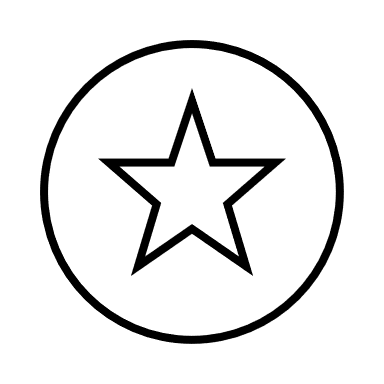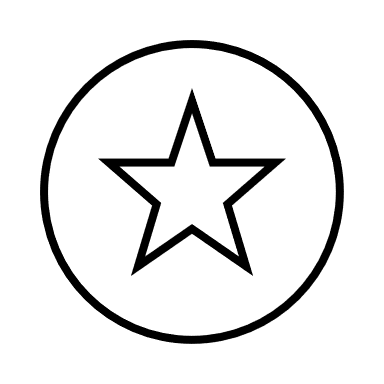 |
| Cissoko, 2005 | 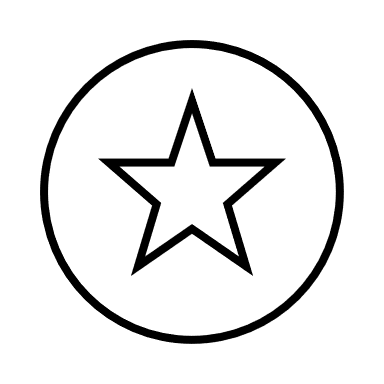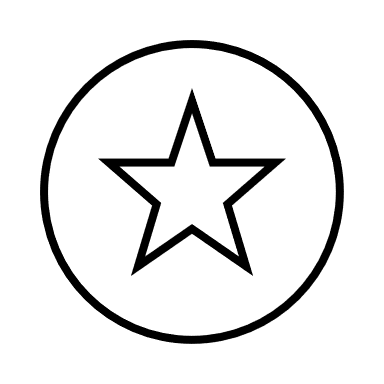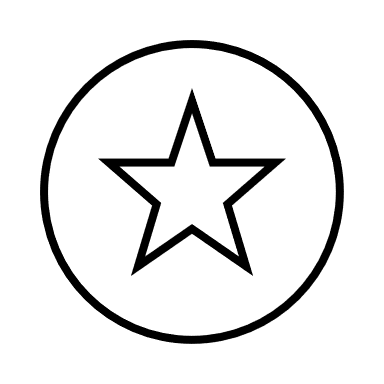 | 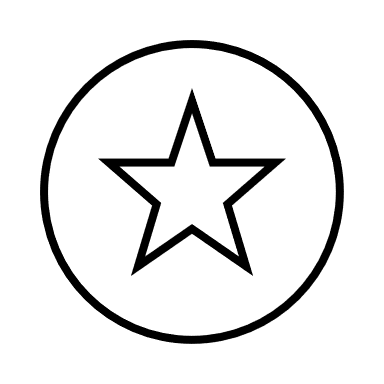 | 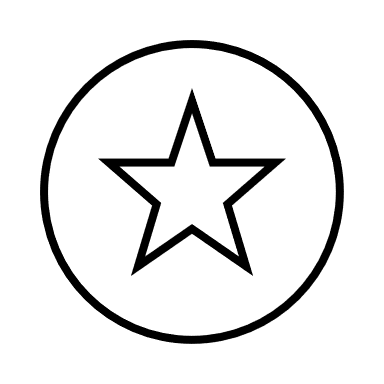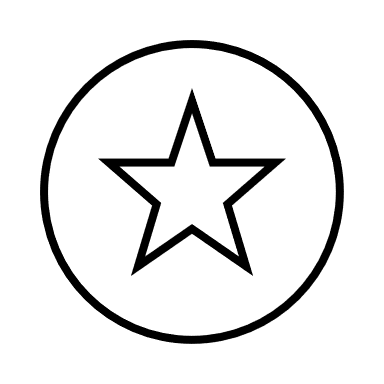 |
| Chera-Aree, 2020 | 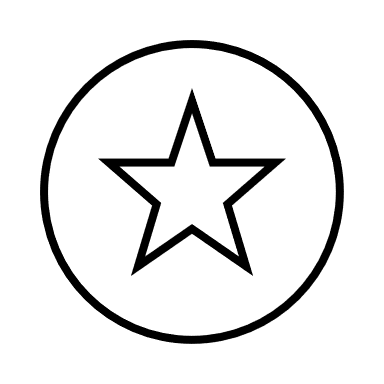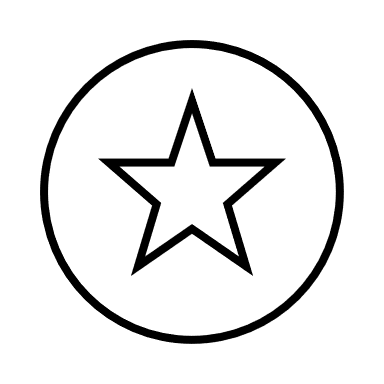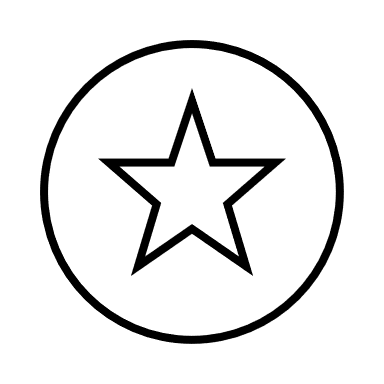 | 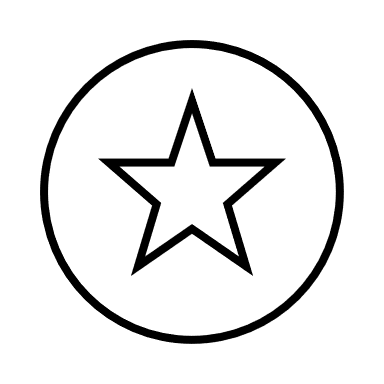 | 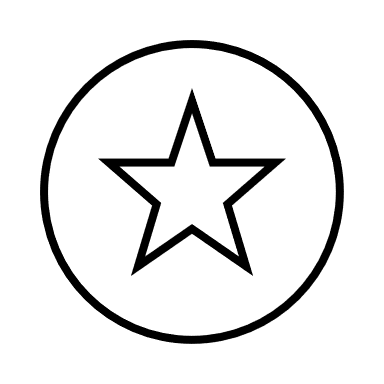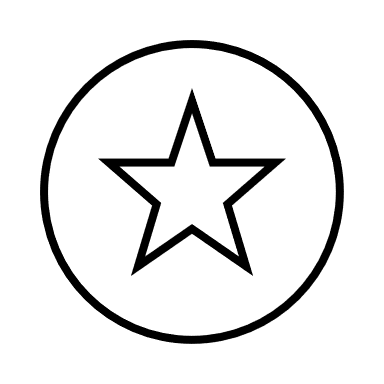 |
| Dumez 1981 | 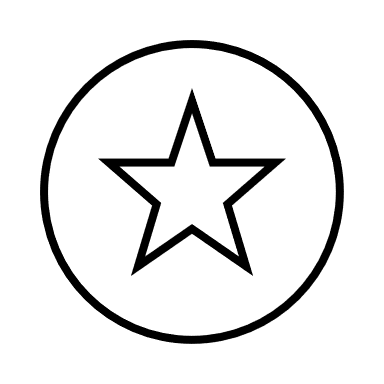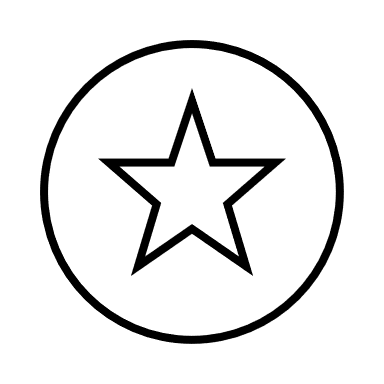 | 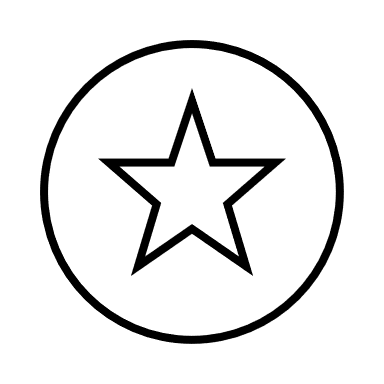 | 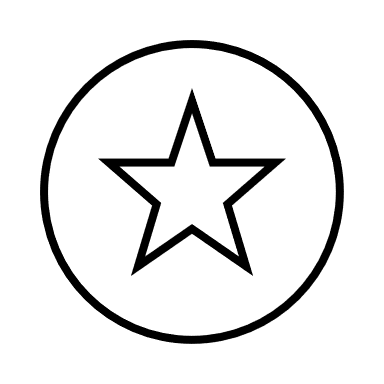 |
| Giannubilo, 2012 | 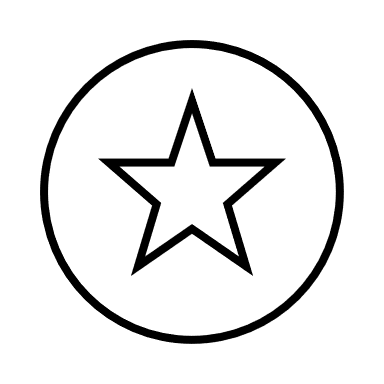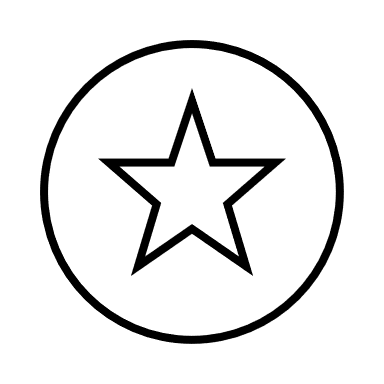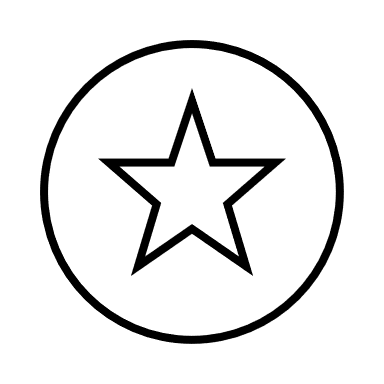 | 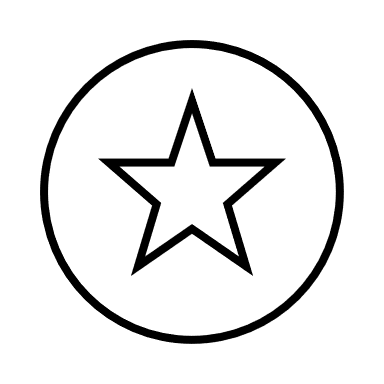 | 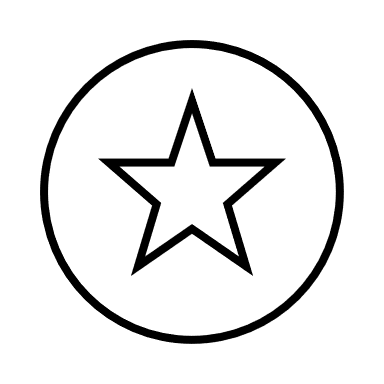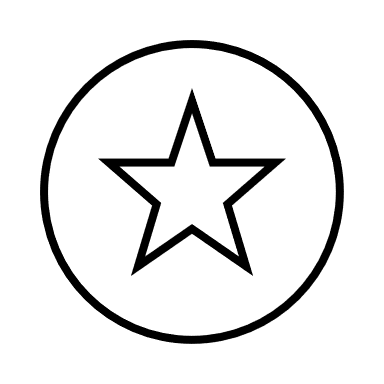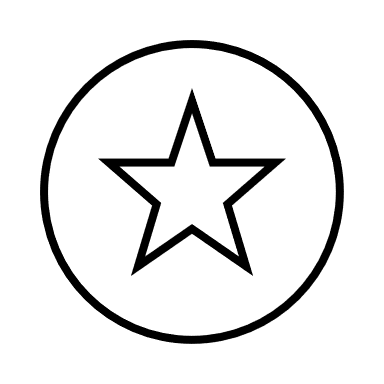 |
| Heida, 2011 | 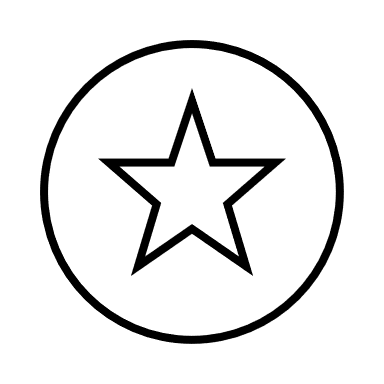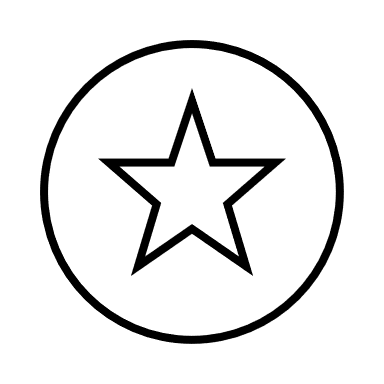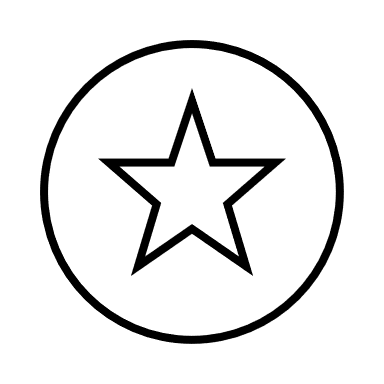 | 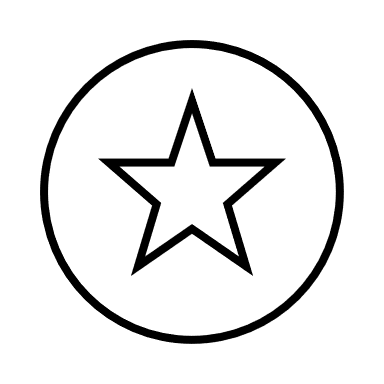 | 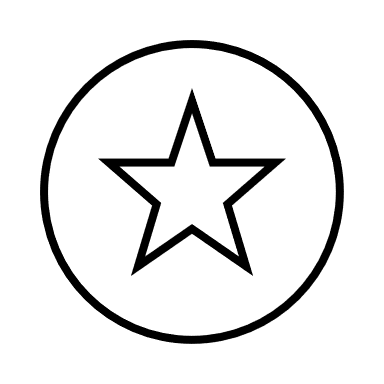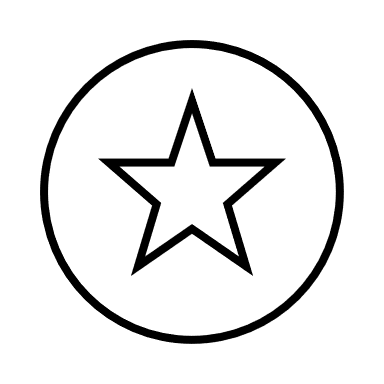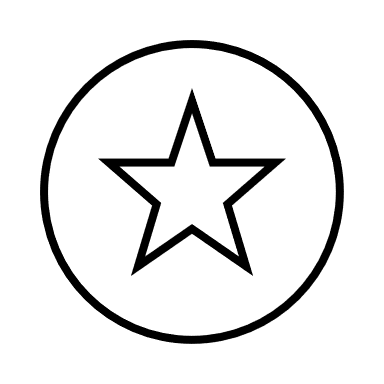 |
| Munshi 1992 | 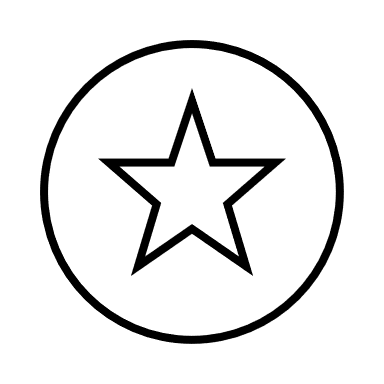 | 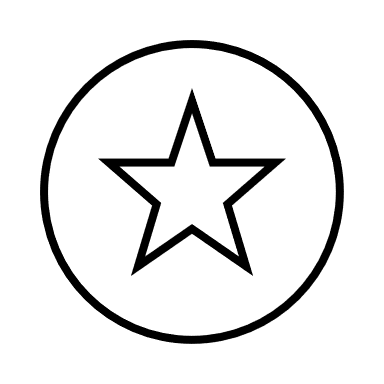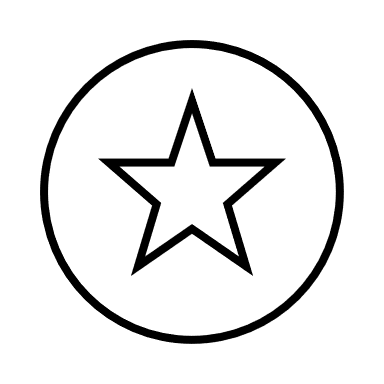 | 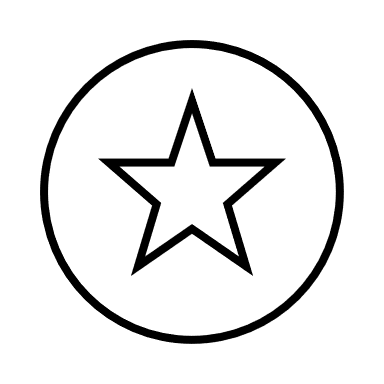 |
| Ping Li, 2021 | 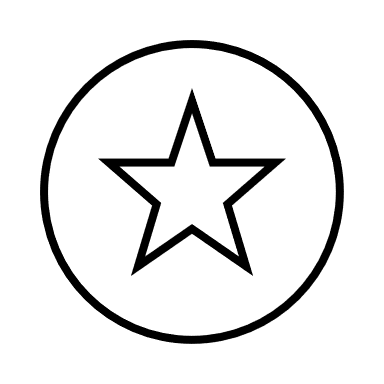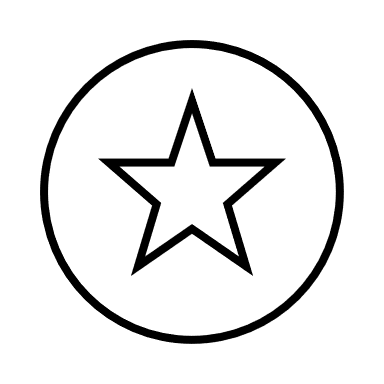 | 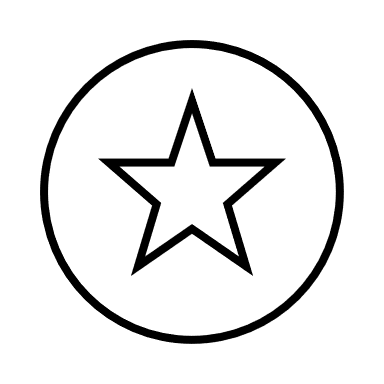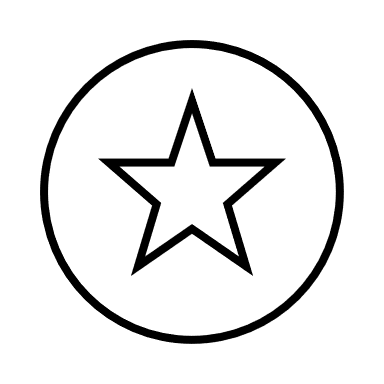 | 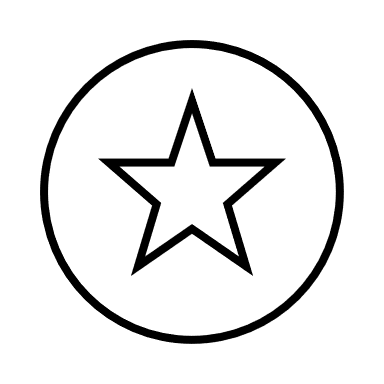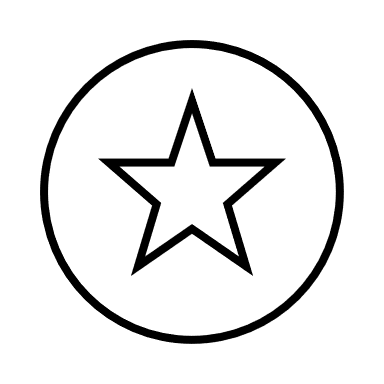 |
| Ritcher, 2016 | 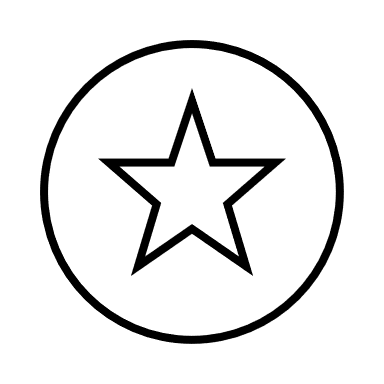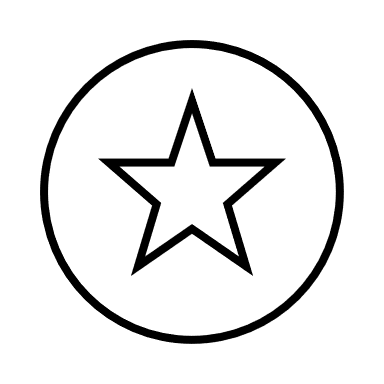 | 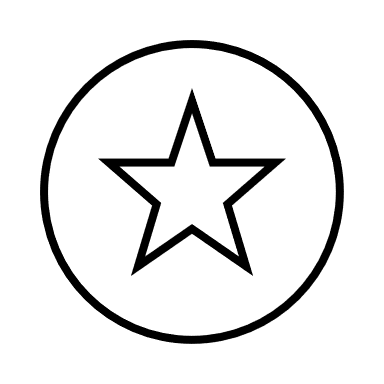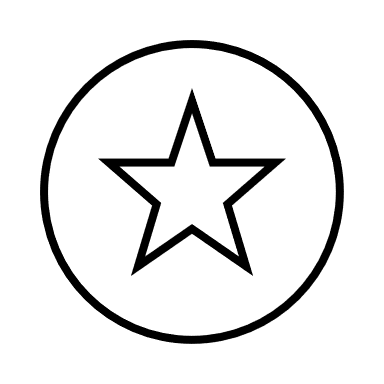 | 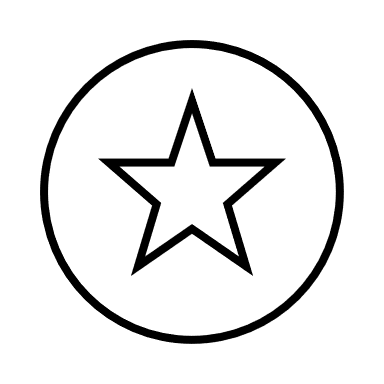 |
| Sandstrom, 1982 | 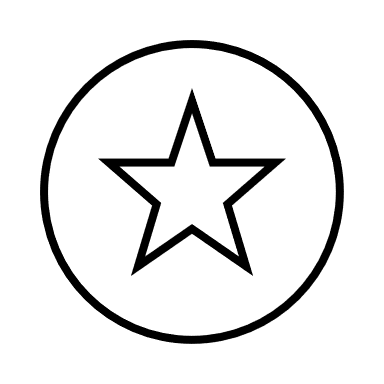 | 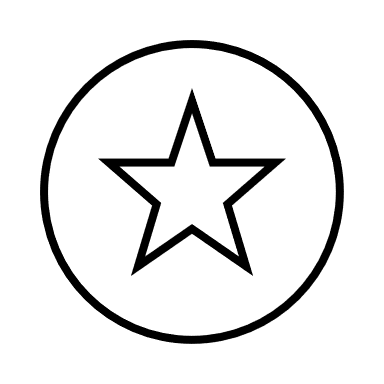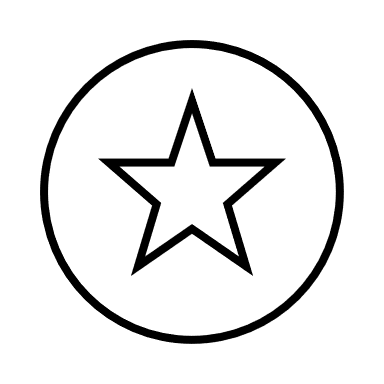 | 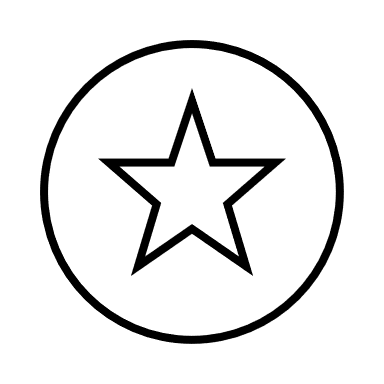 |
| Sharma, 2016 | 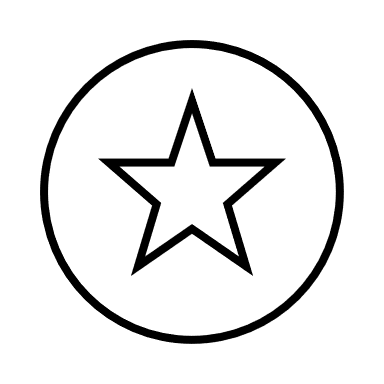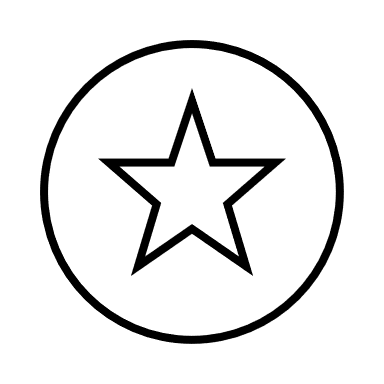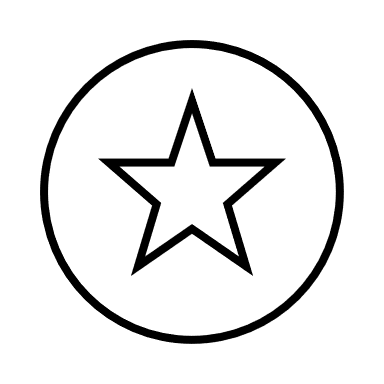 | 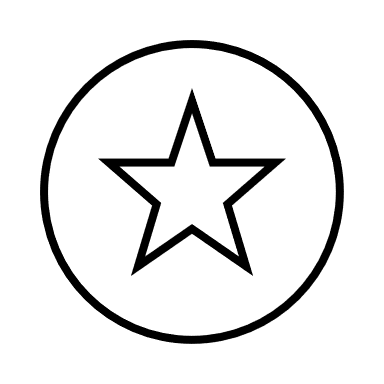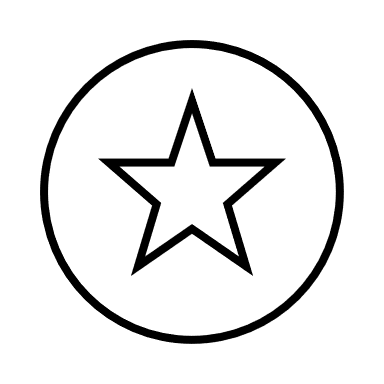 | 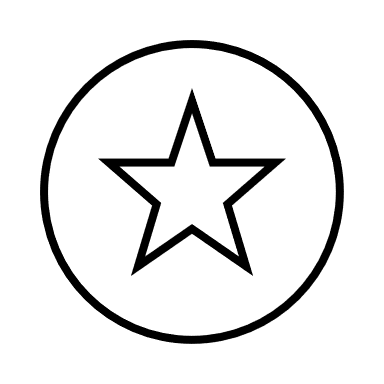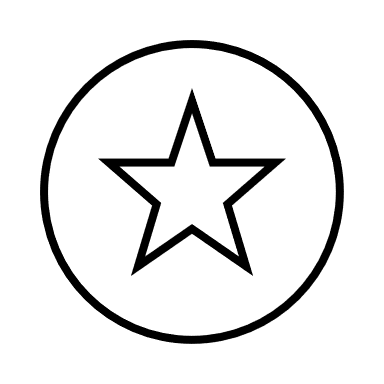 |
| Thewissen, 2016 | 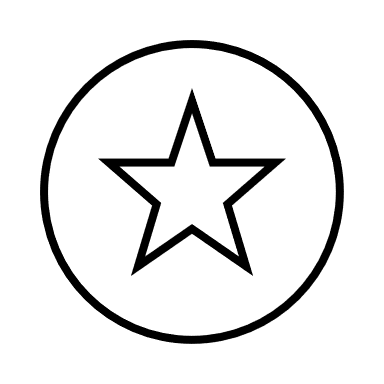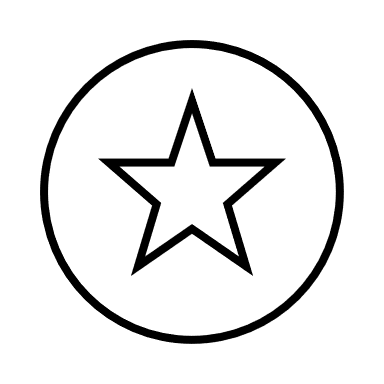 | 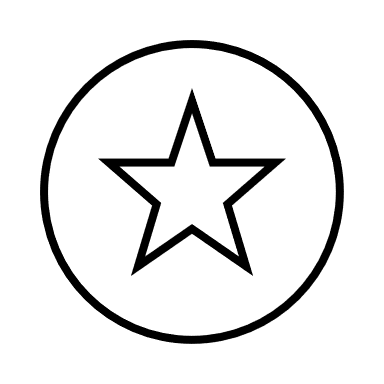 | 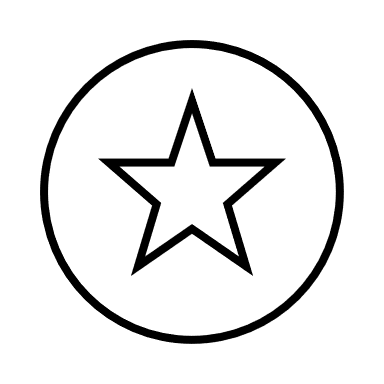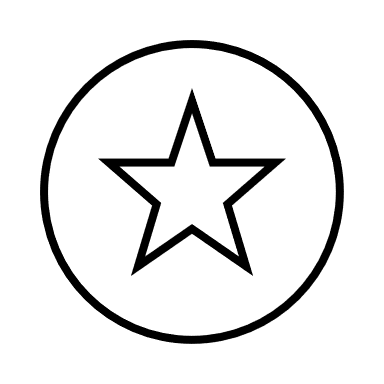 |
| Verhagen, 2013 | 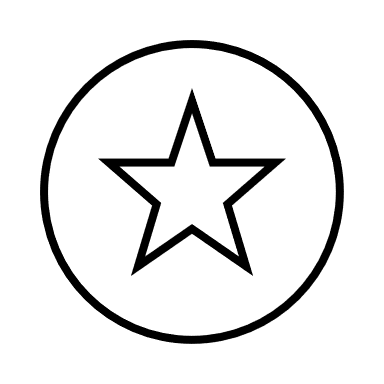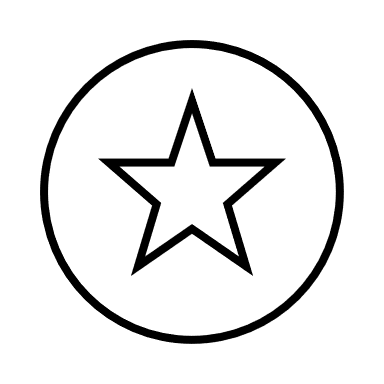 | 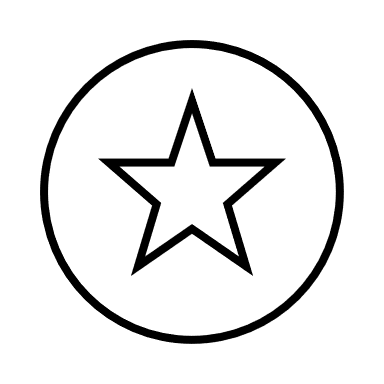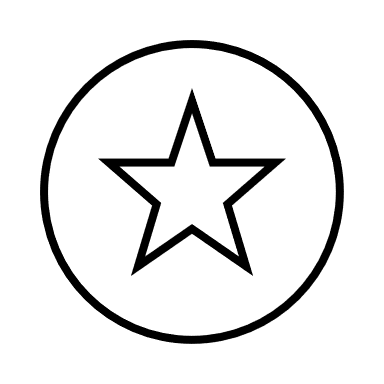 | 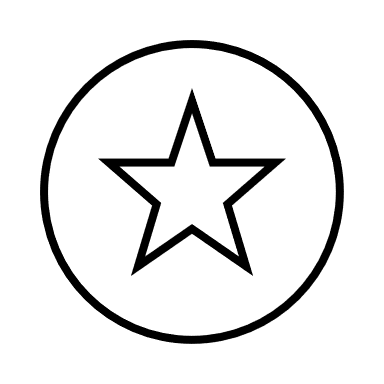 |
| William 1982 | 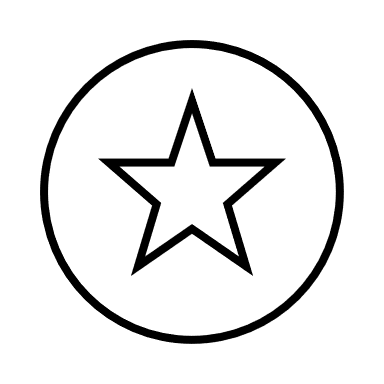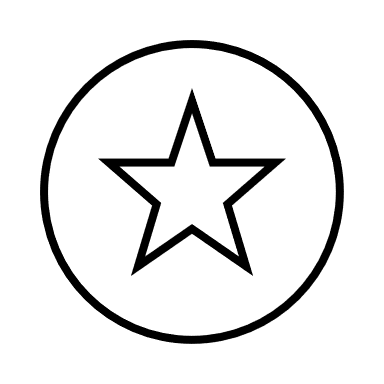 | 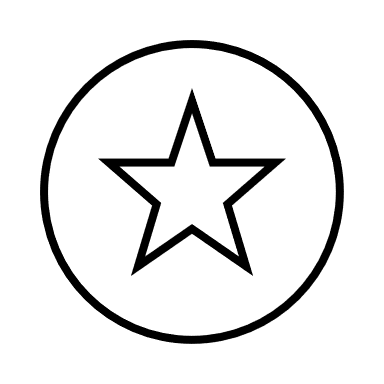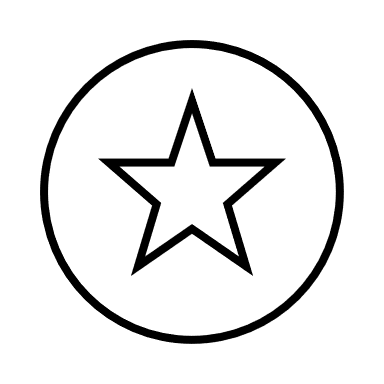 | 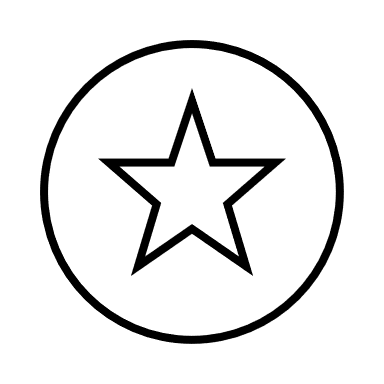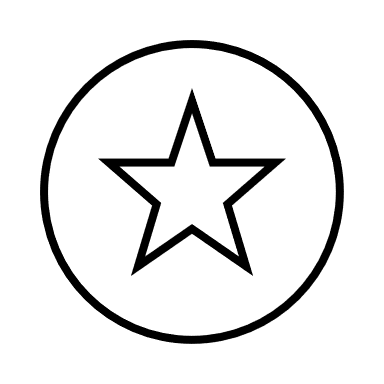 |

* Thresholds for converting the Newcastle-Ottawa scales to Agency for Health Care Research and Quality (AHRQ) standards (good, fair, and poor) are as follows: (i) good quality: 3 or 4 stars in selection domain AND 1 or 2 stars in comparability domain AND 2 or 3 stars in outcome/exposure domain; (ii) fair quality: 2 stars in selection domain AND 1 or 2 stars in comparability domain AND 2 or 3 stars in outcome/exposure domain; and (iii) poor quality: 0 or 1 star in selection domain OR 0 stars in comparability domain OR 0 or 1 stars in outcome/exposure domain.

# Table S7: Risk of bias assessment with the Johanna Briggs Institute Checklist for the included case series*

|  |  |  |  |  |  |
| --- | --- | --- | --- | --- | --- |
|  | Boutroy  1982 | Lurie 1990 | Montan 1993 | Pirhonen 1990 | O’Hare 1980 |
| Were there clear criteria for inclusion in the case series? | 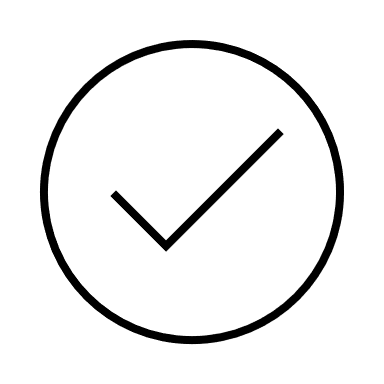 | 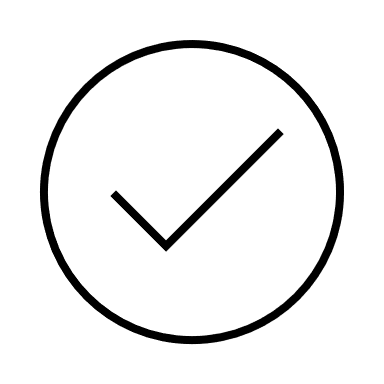 | 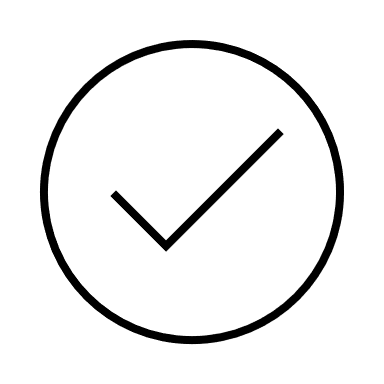 | 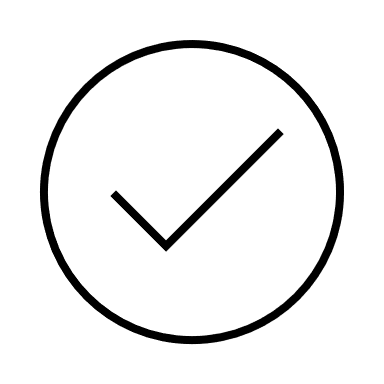 | 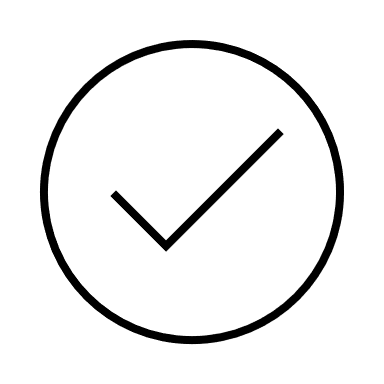 |
| Was the condition measured in a standard, reliable way for all participants included in the case series? | 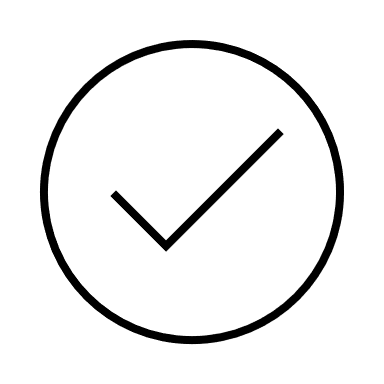 | 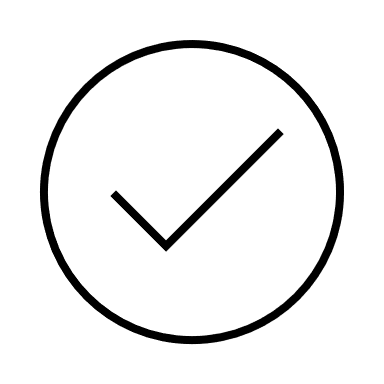 | 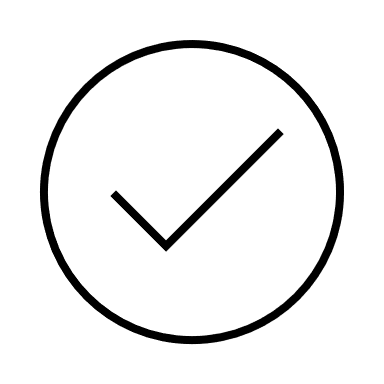 | 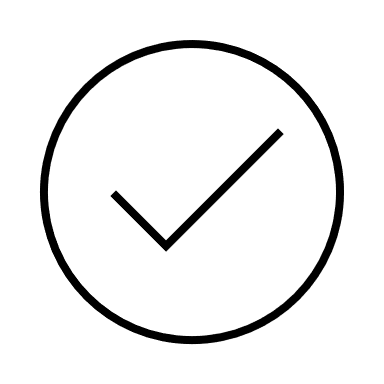 | 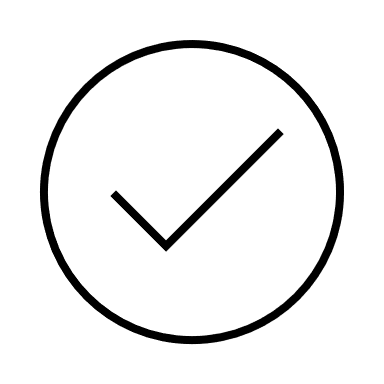 |
| Were valid methods used for identification of the condition for all participants included in the case series? | 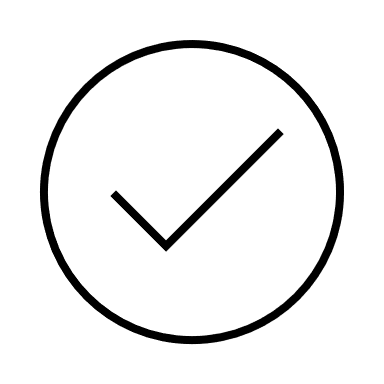 | 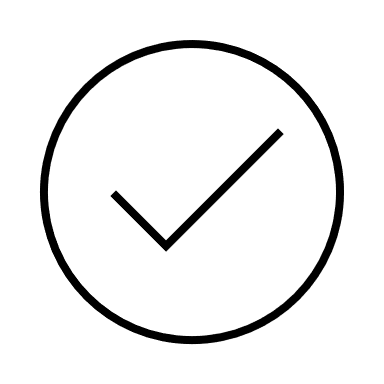 | 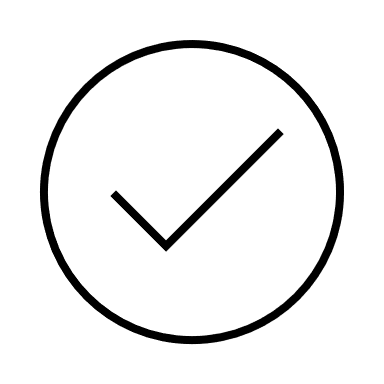 | 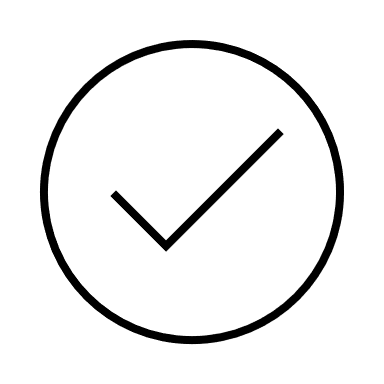 |  |
| Did the case series have consecutive inclusion of participants? |  |  |  |  | No |
| Did the case series have complete inclusion of participants? | No |  |  |  | No |
| Was there clear reporting of the demographics of the participants in the study? | No | No |  |  |  |
| Was there clear reporting of clinical information of the participants? |  |  |  |  |  |
| Were the outcomes or follow up results of cases clearly reported? |  |  |  |  |  |
| Was there clear reporting of the presenting site(s)/clinic(s) demographic information? |  | No |  | No | No |
| Was statistical analysis appropriate? | No |  |  |  |  |
|  |  |  |  |  |  |

# Table S8: Maternal and perinatal outcome rates (median, %) reported in studies of antihypertensive medications that reported outcomes of adverse FHR or neonatal HR effects

|  | RCTs | | Observational studies  (N=29) |
| --- | --- | --- | --- |
|  | Non-severe hypertension (N=30) | Severe hypertension (N=24) |  |
| **Maternal outcomes** |  |  |  |
| Maternal death | 0 [0, 0] (4 studies) | 0 [0, 0] (5 studies) | 1 [0, 1] (1 study) |
| Eclampsia | 0 [0, 0] (6 studies) | 1 [0.5, 1.5] (2 studies) | NA (0 studies) |
| Stroke | NA (0 studies) | 0 [0, 0] (1 study) | NA (0 studies) |
| Cortical blindness | NA (0 studies) | 1 [0.5,1.5] (2 studies) | NA (0 studies) |
| Retinal detachment | NA (0 studies) | 0 [0,0] (1 study) | NA (0 studies) |
| Pulmonary oedema | NA (0 studies) | 0 [0, 0] (1 study) | 1 [0,1] (1 study) |
| Acute kidney injury | NA (0 studies) | NA (0 studies) | NA (0 studies) |
| Liver capsule haematoma or rupture | NA (0 studies) | NA (0 studies) | NA (0 studies) |
| Placental abruption | 0.8 [0, 1.7] (9 studies) | 2.5 [1.1, 3.6] (7 studies) | 1 [0,1)] (1 study) |
| Post-partum haemorrhage | 4.4 [4,6.1] (3 studies) | 9 [4.7, 18.7] (3 studies) | 5 [4,7] (1 study) |
| HELLP | 5 [5, 5] (1 study) | NA (0 studies) | NA (0 studies) |
| Raised liver enzymes | NA (0 studies) | NA (0 studies) | 6 [3,9] (5 studies) |
| Low platelets | 4 [4, 4] (1 study) | NA (0 studies) | 6 [3,9] (5 studies) |
| Maternal ICU admission | NA (0 studies) | 0 [0, 2] (3 studies) | NA (0 studies) |
| Intubation and mechanical ventilation | NA (0 studies) | 0 [0, 0] (1 studies) | NA (0 studies) |
| **Fetal/ neonatal outcomes** |  |  |  |
| Stillbirth | 0 [0, 1.7] (23 studies) | 0 [0, 2.5] (9 studies) | 3 [3,7] (3 studies) |
| Newborn death | 0 [0, 0.9] (22 studies) | 1.8 [0, 4.5] (10 studies) | 2 [2,2] (4 studies) |
| Preterm birth (<37 weeks) | 25 [24.4, 30] (5 studies) | 21.7 [13.9,67.0] (7 studies) | 32 [15, 46] (9 studies |
| Small-for-gestational age | 13.3 [11.2, 13.7] (11 studies) | 9.3 [6.0, 12.1] (4 studies) | 12 [9, 21] (7 studies) |
| SCBU or NICU admission | 10.3 [7, 18] (13 studies) | 12 [10.6,14.8] (7 studies) | 28 [25, 30] (4 studies |
| Neonatal respiratory support | 7.8 [4.3, 7.9] (3 studies) | 18.7 [11.3, 26] (2 studies) | 15.2 [13.7, 16.6] (2 studies) |
| Neonatal seizures | NA (0 studies) | NA (0 studies) | 1 [0,1] (1 study) |

HELLP= Haemolysis Elevated Liver Enzyme and Low Platelet syndrome, NA= not available, RCTs= Randomized Controlled Studies, HR= Heart rate

**Table S9**: Antihypertensive drug-vs-drug direct and indirect evidence, and inconsistency indices for the main outcomes*

| **ANTIHYPERTENSIVE DRUG VS. DRUG COMPARISONS** | | **NETWORK meta-analysis** | | | |
| --- | --- | --- | --- | --- | --- |
| **Treatment** | **Comparator** | **Direct evidence**  **OR (95%CI)** | **Indirect evidence**  **OR (95%CI)** | **Overall OR**  **(95% CI)** | **p value**  **inconsistency*** |
| **Adverse FHR effects** | |  |  |  |  |
| **Non-severe hypertension** | |  |  |  |  |
| Labetalol | Placebo/none | 2.18 (0.61–12.40) | 0.24 (0.03–2.70) | 1.27 (0.38–5.65) | 0.09 |
| Other BB | Placebo/none | - | - | 1.90 (0.19, 16.0)ǂ | - |
| Methyldopa | Placebo/none | 4465.79 (0.28–339256132067211136.0) | 1.69 (0.33–9.77) | 1.99 (0.46–11.67) | 0.19 |
| CCB | Placebo/none | 0.41 (0.04–2.70) | 7.46 (1.23–79.33) | 1.88 (0.40–8.78) | 0.028 |
| Mixed | Placebo/none | - | - | 1.70 (0.30, 8.70)ǂ | - |
| Labetalol | Methyldopa | 0.64 (0.14–2.60) | 0.61 (0.05–8.63) | 0.64 (0.19–2.00) | 0.98 |
| Methyldopa | Other BB | 2.15 (0.18–27.80) | 8.96 (0.49–253.30) | 3.67 (0.65–25.64) | 0.41 |
| CCB | Methyldopa | 0.73 (0.09–6.23) | 1.11 (0.11–5.96) | 0.93 (0.20–3.28) | 0.75 |
| Mixed | Methyldopa | - | - | 3.30 (0.37, 34.0)ǂ | - |
| CCB | Labetalol | 5.97 (0.78–46.19) | 0.71 (0.14–2.84) | 1.47 (0.32–5.32) | 0.07 |
| CCB | Other BB | - | - | 0.29 (0.05, 1.90)ǂ | - |
| CCB | Mixed | - | - | 0.32 (0.03, 3.30)ǂ | - |
| Labetalol | Other BB | - | - | 0.42 (0.06, 3.20)ǂ | - |
| Mixed | Other BB | - | - | 0.91 (0.06, 15.0)ǂ | - |
| Labetalol | Mixed | - | - | 0.47 (0.06, 3.60)ǂ | - |
| **Severe hypertension** | |  |  |  |  |
| Labetalol | Placebo/none | 2.42 (0.02–332.33) | 4.19 (0.02–654.32) | 3.05 (0.10–79.91) | 0.87 |
| CCB | Methyldopa | - | - | 2.90 (0.04, 4600)ǂ | - |
| CCB | Placebo/none | 2.83 (0.02–277.28) | 1.60 (0.01–369.36) | 2.15 (0.08–58.06) | 0.87 |
| CCB | Labetalol | 0.63 (0.04–13.98) | 0.82 (0.03–15.40) | 0.71 (0.10–5.50) | 0.89 |
| CCB | Hydralazine | 2.80 (0.15–49.52) | 2.75 (0.11–97.88) | 2.73 (0.37–22.44) | 0.99 |
| Hydralazine | Labetalol | 0.26 (0.04–1.68) | 0.25 (0.01–16.50) | 0.26 (0.05–1.26) | 0.98 |
| **Adverse neoHR effects** | |  |  |  |  |
| **Non-severe hypertension** | |  |  |  |  |
| **Labetalol** | Placebo/none | - | - | 1.40 (0.53, 3.40)ǂ |  |
| **CCB** | Placebo/none | - | - | Cannot estimate |  |
| **Other BB** | Placebo/none | 1.44 (0.28–7.36) | 2.90 (0.14–59.59) | 1.51 (0.54–5.18) | 0.64 |
| **CCB** | Hydralazine | - | - | Cannot estimate |  |
| **CCB** | Labetalol | - | - | Cannot estimate |  |
| **Hydralazine** | Labetalol | - | - | Cannot estimate |  |
| **Other BB** | Methyldopa | - | - | 1.90 (0.00002, 190000)ǂ | - |
| **Other BB** | Labetalol | - | - | 0.48 (0.16, 1.10)ǂ | - |
| **Mixed** | Hydralazine | - | - | Cannot estimate | - |

BB=beta-blockers, CCBs=calcium channel blockers, CI=credible interval, I^2^=inconsistency index, NA=not applicable, NE=Not estimable, OR=odds ratio

* This evaluates consistency between direct and indirect evidence.

ł Can only be estimated when there is at least one direct comparison and one indirect path.

ǂ Network could not be split into direct and indirect pathways due to a lack of direct evidence, and indirect path lengths >2 that did not allow for stable network ‘splitting’.

**Figure S1**: Forest plots of RCTs assessing the impact of antihypertensives (vs. placebo/no therapy) on adverse FHR effects, among women treated for non-severe hypertension – SENSITIVITY ANALYSIS for Figure 2a (excluding El Guindy *et al*. [ref])

Legend: CCB (calcium channel blocker), FHR (fetal heart rate), RCTs (randomised controlled trials)

**Figure S2**: Forest plots of RCTs assessing the impact of labetalol or other beta-blockers (vs. other antihypertensives) on adverse FHR effects, among women treated for non-severe hypertension – SENSIVITY ANALYSIS for Figure 2c (excluding Jannet *et al*. [ref])

Legend: CCB (calcium channel blocker), FHR (fetal heart rate), RCTs (randomised controlled trials)

**Figure S3**: Forest plots of RCTs evaluating the impact of antihypertensive therapy (vs. CCBs) on adverse FHR effects, among women with severe hypertension – SENSITIVITY ANALYSIS for Figure 3c (excluding Easterling *et al*. [ref] and Scardo et al. [ref])

Legend: CCB (calcium channel blocker), FHR (fetal heart rate), RCTs (randomised controlled trials)
